# How culturally wise psychological interventions can help reduce poverty

**DOI:** 10.1073/pnas.2505694122

**Published:** 2025-11-13

**Authors:** Catherine C. Thomas, Patrick Premand, Thomas Bossuroy, Soumaila Abdoulaye Sambo, Hazel Rose Markus, Gregory M. Walton

**Affiliations:** ^a^Department of Psychology, University of Michigan, Ann Arbor, MI 48109; ^b^Organizational Studies, University of Michigan, Ann Arbor, MI 48109; ^c^Department of Psychology, Stanford University, Palo Alto, CA 94305; ^d^Development Impact, Development Economics, World Bank, Washington DC 20433; ^e^Social Protection and Labor, World Bank, Washington DC 20433; ^f^Groupe de Recherche, d’Etudes et d’Action pour le Développement, Niamey 10380, Niger; ^g^Department of Psychology, Stanford SPARQ, Stanford CA 94305

**Keywords:** poverty, behavioral science, psychology, culture, economic empowerment

## Abstract

Poverty is characterized by multidimensional economic, social, and psychological constraints that undermine people’s agency to pursue new opportunities and shape their life outcomes. How can interventions best support the agency of low-income individuals and, in so doing, boost poverty-reduction efforts? We theorize and find that agency interventions are effective when designed to be “culturally wise,” i.e., attuned to the model of agency predominant in a cultural context. Focusing on low-income women in rural Niger, Study 1 finds that local mental models of economic success primarily reflect interdependence, grounded in relational factors like advancing social harmony, respectfulness, and collective benefits. As evidenced by data from a United States sample, this contrasts with a more independent model common in the West grounded in personal factors like self-initiative. Study 2 finds empirical support for relational factors (e.g., subjective social standing) in addition to self-oriented personal factors (e.g., self-efficacy) as mechanisms of women’s economic advancement in a highly effective multifaceted poverty reduction program. Study 3 reports a field experiment with program participants (n = 2,628) to compare a Western-derived *personal agency* intervention and a culturally wise *relational agency* intervention each to a control. Only *relational agency* caused significant improvements in economic outcomes over 12 mo, as well as in some personal and relational outcomes. By contrast, *personal agency* showed limited effects, shifting only personal outcomes. These findings reveal the promise of research at the intersection of social and cultural psychology, behavioral science, and development economics to help address global poverty.

## Significance

Efforts to alleviate poverty often overlook the role of psychosocial factors. Three interdisciplinary studies explore a “culturally wise” approach to supporting the agency of women in poverty. In resource-constrained rural Niger, this entailed attending to an interdependent model of agency grounded in social harmony, respect, and collective advancement, as compared to relying solely on a Western independent model grounded in factors like self-initiative. A field experiment showed that an intervention emphasizing this *relational agency* significantly improved women’s economic outcomes compared to control, while a prototypical intervention emphasizing *personal agency* did not. Together, these interdisciplinary studies offer a blueprint for considering cultural variation in agency and the promise of doing so to help reduce global poverty across diverse cultural contexts.

“Hannu daya baya daukan jinka/
  One hand cannot lift a hut.” — Hausa proverb (1)

Everywhere poverty means a lack of financial resources. Yet living in poverty also influences who you are, what others are likely to think about you, and what, if anything, you can do to navigate these circumstances and realize your aspirations (2, 3). Despite their profound consequences for behavior, the psychosocial challenges of poverty have been too often a policy blind spot. A small but emerging literature (4–11) is revealing the potential of scalable psychosocial interventions to support people’s capacity to act and pursue pathways out of poverty, particularly when paired with economic opportunity. What makes psychosocial interventions to support people’s agency effective or ineffective in reducing poverty? We posit here that their potential depends in part on the extent to which they are attuned to local models of agency predominant in a given cultural context.

We define agency as the capability to act and to shape one’s life outcomes (12, 13) in ways that feel good, moral, and fitting for one’s cultural context (13, 14). Research in social and cultural psychology finds that models of agency are as diverse as the cultural and socioeconomic contexts that people seek to navigate (15–17). These contexts vary both across and within geographic regions. High-income Western contexts with material abundance, formal and impersonal institutions, and high levels of cultural individualism (18, 19) afford and foster more independent models of agency. These models entail self-directed, autonomous action toward the advancement of personal interests and goals.

By contrast, in low-income contexts in the Global South, where most of the world’s population resides, people often live in tight knit, enduring webs of social connection, mutual support, and obligation. These contexts tend to afford and foster a variety of more interdependent models of agency. Broadly, these models entail greater responsiveness to others, role requirements, and social obligations. As people rely on their social networks to cope with resource scarcity and to access information and opportunities, their capacity to realize their aspirations often depends on support from others (20). Such interdependent agency emphasizes ongoing coordination with others for collective advancement (2, 16, 20–23). As Ghanaian cultural and social psychologist Stephen Adjei writes, “in the African epistemic worldview the person is ontologically part of the social firmament”; there, “negotiated agency involves joint decision-making and being responsive to expectations and demands of relational others in a network of interconnectedness” (24).

Both models of agency can be identified in most cultural contexts and, in some populations, the models can be quite intertwined (25). For example, in the case of sub-Saharan Africa, emerging research identifies a particular variety of interdependence in which personal ambition is primarily enacted in the service of the in-group and its collective advancement (8, 26, 27). In this case, interdependent motivation may activate, or allow for, personal striving, particularly when this striving strengthens one’s relationships. As an example of this cultural logic, if you feel that others in your household and community hold you in high regard, you may feel more self-efficacious to start a new business. You may also feel more motivated and optimistic if you feel that the business could, in turn, benefit your group and enhance your social standing.

Implicitly or explicitly, all programs and interventions that address agency are grounded in context-specific models of agency; they invite people to pursue goals in a particular way. People are most likely to be receptive when the invitations feel like a fit or match for who they are, and want to become, in a given context (21, 23, 28). Over 90% of psychological research to date has been based on high-income, individualistic Western populations (29). Fitting with this geographic focus, a prototypical, independence-oriented motivational intervention from Western contexts prompts individuals to set personal goals, to pursue them through self-initiative, and to plan self-regulation strategies to cope with setbacks (30, 31). One large-scale cross-cultural experiment found this type of intervention boosted academic achievement in the United States, consistent with past studies, yet not in India or China (32). Follow-up surveys revealed signs of insufficient cultural adaptation, specifically to address competing relational considerations (26): Students in India, as compared to those in the United States, saw personal academic goals as secondary to fulfilling social obligations (e.g., chores) and relational goals (e.g., not disappointing family) (see also refs. 33 and 34).

Psychologically wise interventions are those that seek to empower people by attending to how they make sense of themselves, their agency, and the opportunities they seek to navigate (35). Culturally wise interventions further attend to cultural variation in these processes intentionally and directly. They are wise to the fact that people are enculturated actors whose models of agency, among other psychosocial characteristics, systematically vary across cultural contexts (15, 16, 36). One laboratory experiment provides initial, suggestive evidence for the importance of culturally wise approaches for economic empowerment. Thomas et al. (8) offered residents in low-income communities in Nairobi, Kenya small cash transfers while randomly varying the narrative advanced by the donor organization. Compared to a status quo aid narrative (control), an interdependent agency narrative—representing the cash as a way to support one’s contributions to community and collective advancement—significantly increased recipients’ interest in building their business skills and improved relational outcomes (e.g., less perceived stigma). Its effects did not significantly differ from a second agency narrative representing cash as a way to support independent agency and goals like self-reliance and self-advancement. This independent agency narrative, however, showed limited effects compared to the control, significantly improving only personal outcomes such as self-efficacy.

**Fig. 1.**
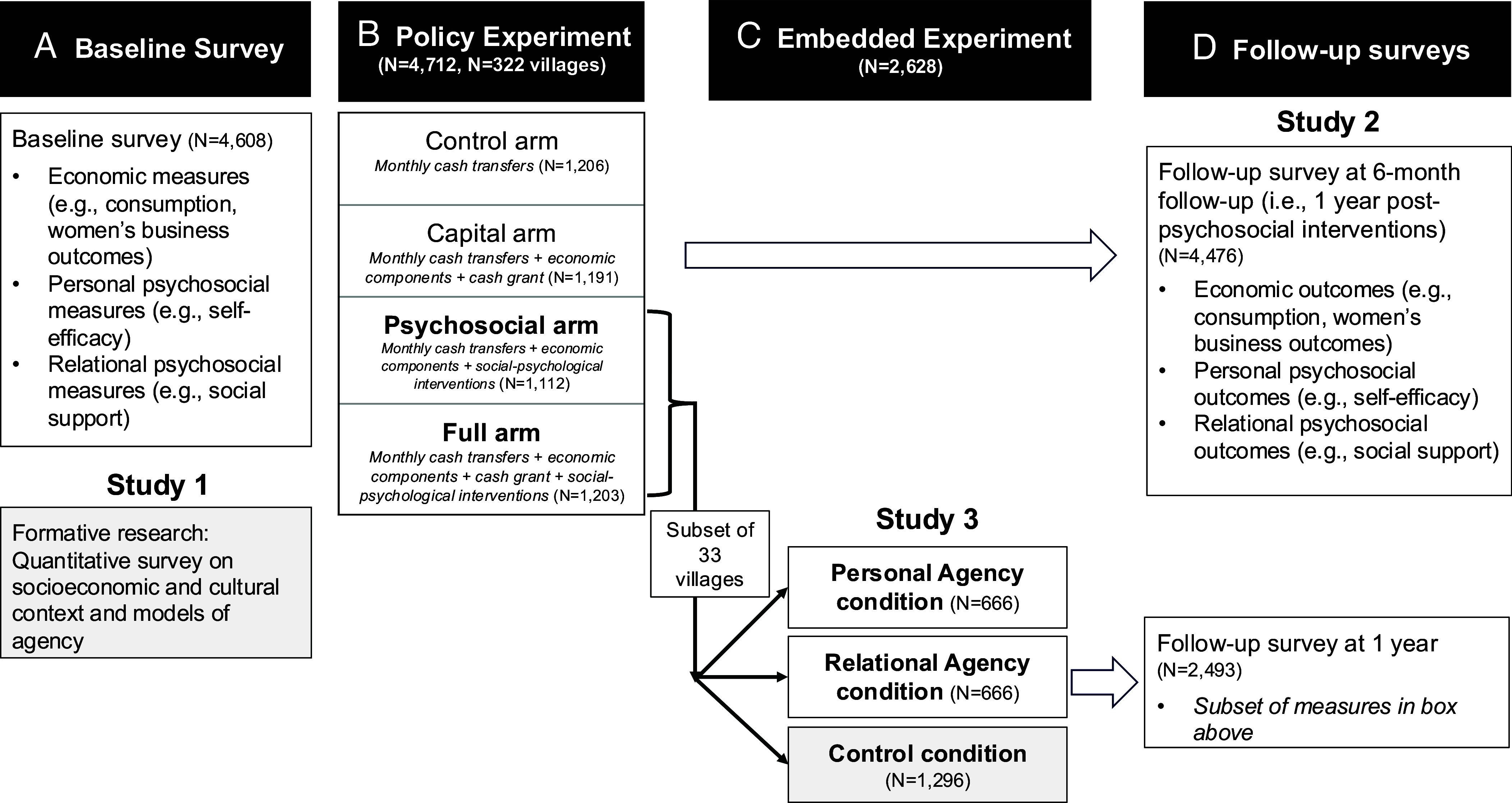
Design of Studies 1–3. This figure depicts the sources of data for each study and chronology of research activities. Baseline data (*A*) was collected as part of the policy experiment (*B*), in which the experiment in Study 3 was embedded (*C*); follow-up surveys were conducted approximately 1 year after delivery of the psychosocial interventions (*D*). The total number of Sahel Adaptive Social Protection program recipient households was 22,500 across the 17 communes with the highest poverty rates nationwide. Study 1 used data from the baseline of the policy experiment (*A*) and control group of the embedded experiment (*C*). Study 2 included data from the four-arm policy experiment (*B* and *D*), whose sample included a random subset (n = 4,712) of all program participants and was randomized at the village level. Study 3 was an individual-level embedded experiment (*C*), whose sample included all program participants in a nonrandom subset of 33 villages in six communes (n = 2,628) (*SI Appendix,* Fig. S1), including but not limited to those participants randomly sampled for the policy experiment. This research was conducted through a continuous engagement with the Niger Safety Nets Unit and the World Bank’s Sahel Adaptive Social Protection program over the period 2017 to 2022.

Here, in three studies in Niger, one of the lowest-income nations in the world, we ask the following questions: Will interventions grounded in a more Western, independent model of agency be sufficient to help reduce poverty among rural women in Niger? Or will they need to be designed to be “culturally wise” to local models of agency? To address these questions, we report three sets of results (Fig. 1). Study 1 (descriptive) seeks to understand models of women’s economic success in rural Niger. It finds an interdependent model—grounded in the advancement of social harmony and respect—to be predominant. By contrast, a US sample assumed an independent model, predicting factors like self-initiative and future-orientation to be relatively more important. Study 2 (mechanisms analysis) provides empirical support for the importance of relational mechanisms (such as subjective social standing) in addition to self-oriented personal mechanisms (such as self-efficacy) in contributing to women’s economic gains from an effective multifaceted poverty reduction program that included agency interventions. Study 3 (experimental) assesses the separable impacts of independent and interdependent models of agency through a supplemental individual-level intervention embedded in the policy experiment (n = 2,628). As predicted, a more culturally wise *relational agency* intervention—emphasizing collective advancement and social harmony—significantly improved women’s economic outcomes 1 y later compared to a control, as well as some personal and relational outcomes. By contrast, an independence-oriented *personal agency* intervention, which emphasized self-advancement and personal initiative, produced limited effects compared to the control, significantly improving personal outcomes but neither relational nor economic outcomes.

## Study 1: Local Models of Agency.

This formative research sought to understand the predominant models of agency among women in rural Niger, specifically which qualities and behaviors women viewed as valued, normative, and effective for advancing their economic mobility. We developed quantitative measures to elicit women’s models of agency based on interviews and focus groups. We coupled these results with baseline survey data from the policy experiment (Fig. 1) on the socioeconomic and cultural context to build interpretive power and to understand these models of agency in relation to the affordances and constraints of the local context (37).

Women in our study were from the lowest income households in one of the lowest income countries in the world. Over 90% had never been to school and few were literate. They lived in remote areas, an average of 73 min by foot from the nearest market. In formative research, women reported encountering strangers, people they did not know, approximately twice a month. In these remote, rural areas with little access to formal education and technology, women are highly dependent on other people in their village and on word-of-mouth for economic information, opportunities, and resources.

Across different survey measures describing the cultural context, we found that interdependence was broadly practiced and valued. For example, in the baseline survey of the policy experiment, 90% of women agreed or strongly agreed that “when making a decision, it is important to take into account the opinions of other members of your community,” and 80% agreed or strongly agreed that “it is your duty to take care of others in your village even if you must sacrifice yourself.” Data collected from the control group in Study 3 revealed that 86% of respondents preferred that households in the village develop together versus independently.

**Fig. 2.**
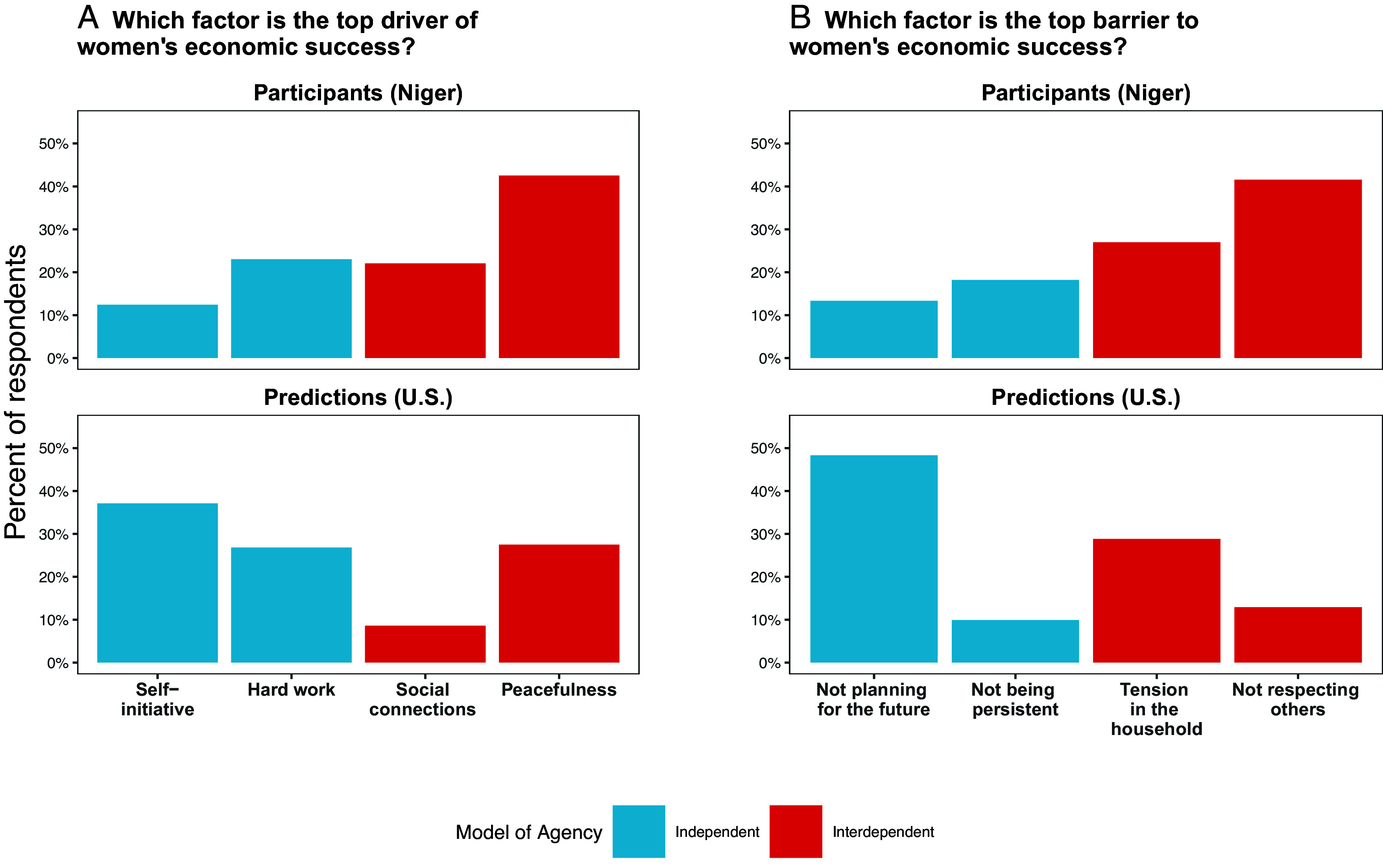
Mental models of women’s agency according to low-income women participants in rural Niger and predictions of a US sample. Mental models assessed perceived drivers of women’s economic success (*A*) and perceived barriers to success (*B*). Responses for Niger sample (top row) were collected among the control group of the embedded experiment in the follow-up survey (n = 1,216). Responses for predictions (bottom row) were collected in an online convenience sample in the United States (n = 302).

Second, these interdependent tendencies were seen as useful and effective. To assess participants’ mental models of women’s economic success (i.e., what they perceive to be key determinants), we asked women to rank the importance of four psychosocial factors as drivers of women’s economic success and four as barriers (Fig. 2). Factors reflecting an independent model of agency were those common in Western settings and embedded in typical self-regulation interventions and other entrepreneurship programs, including the presence (or absence) of self-initiative, hard work, future orientation, and persistence (31, 38–40). Factors reflecting a more interdependent model of agency were derived from our formative qualitative interviews with local respondents. These included interpersonal tension, respect of others, social connections, and peacefulness. Notably, we consider peacefulness to be a feature of interdependent agency given its inextricable grounding in both inner peace and social harmony; research in West Africa finds it to serve as “a signal that one has met social expectations” (41).

As shown in Fig. 2*A*, Nigerien women identified peacefulness as the top factor driving women’s economic success, followed by hard work, social connections, and, lastly, self-initiative. Taken together, they ranked the interdependent factors as significantly more important than the independent factors (χ^2^ (1, *n =* 1,216) = 103.06, *P* < 0.001). Similarly, when asked to rank factors that could serve as barriers to women’s economic success, Nigerien women participants again ranked the interdependent factors—a lack of respect for others and interpersonal tension—as significantly more important than the independent factors—not planning for the future and a lack of persistence (χ^2^ (1, *n =* 1,216) = 166.53, *P* < 0.001) (Fig. 2*B*).

Separately, we collected predictions from a US online convenience sample (n = 302), gathered ex post through the CloudResearch platform, to reveal Western assumptive foundations of agency. We interpret these predictions as projections of factors seen as practiced, valued, and effective according to one’s experiences in one’s own cultural context, i.e., a US context. Consistent with the Western psychological literature grounded in independence (40), when asked to predict which of these factors would be the most important for the economic success of low-income women in Niger (Fig. 2*A*), US respondents identified self-initiative as most important—the factor that Nigerien women selected least. Taken together, US respondents ranked independent factors as more important than interdependent factors (χ^2^ (1, *n =* 302) = 23.36, *P <* 0.001). In relation to barriers to success (Fig. 2*B*), US respondents again ranked independent factors as more important than interdependent factors (χ^2^ (1, *n =* 302) = 8.28, *P* = 0.004). They predicted the top barrier would be not planning for the future, the factor selected least by Nigerien women.

These results suggest that, in this context, successful goal pursuit may critically involve treating others with respect and maintaining social harmony, for instance, to influence household economic decisions or to recruit critical social capital and support. While most Nigerien participants selected factors reflecting an interdependent model, a meaningful proportion (approximately 30%) selected factors reflecting an independent model. These findings suggest that both models are available and relevant in this population but that an interdependent model of agency is predominant and foundational. Our findings are also consistent with the possibility that personal processes such as self-initiative can be in the service of and contribute to the realization of interdependent goals and relational expectations.

Next, we empirically assess these local mental models through examining the roles of psychosocial processes, both relational and personal, in women’s economic success.

## Study 2: Relational and Personal Mechanisms of Poverty Reduction.

Through which psychosocial processes might effective agency interventions, coupled with economic support, contribute to women’s upward economic trajectories in this cultural context? In Study 2, we explored this question by probing the mediating role of relational and self-oriented personal factors in the economic gains of women participants in a highly effective multifaceted poverty reduction program in rural Niger. We expected effective agency in this cultural context to reflect an integration of relational and personal processes that together enable economic empowerment in practice—for instance, feeling supported and respected by others alongside a sense of personal efficacy.

This randomized policy experiment tested different variations of a multifaceted poverty reduction program and assessed their effects on an extensive battery of psychosocial measures. The program variations were compared to a control group that received a more basic type of support (small monthly cash transfers). All program variations included a package of economic interventions (e.g., small monthly cash transfers, savings groups, micro-entrepreneurship training) plus the addition of either psychosocial interventions (“Psychosocial” arm), a large additional cash grant (“Capital” arm), or both (“Full” arm) (Fig. 1). Bossuroy et al. (6) found that all versions produced meaningful gains in poverty reduction, with the “Psychosocial” package in particular yielding one of the most *cost*-effective such designs documented in the literature to date. The two psychosocial interventions sought to boost women’s agency. The first was a community event that displayed a film about a woman named Amina who pursues a new business in the face of a drought. This film was followed by a group discussion that addressed community aspirations, values, and norms about women’s entrepreneurship. The second intervention was a 1-wk training on life skills like goal setting, problem solving, and decision-making and which also used the film to model these skills. These two interventions reflected features of both interdependent and independent models of agency, including social respect and collaboration as well as personal proactivity and persistence.

While this dual-model intervention design does not allow us to distinguish the impacts of interdependent and independent models of agency (which is the aim of Study 3), it does allow for the exploration of a range of possible psychosocial mechanisms underlying this successful program. Toward this goal, we conducted new secondary analyses on other-oriented relational measures, self-oriented personal measures, and women’s business revenues with the policy experiment dataset (6). These measures were assessed in a follow-up survey conducted approximately 6 mo after the end of the multifaceted program’s implementation and 1 y after the implementation of the psychosocial interventions in particular. These measures were informed by Study 1 and other formative research. Relational factors included measures of social capital (e.g., social and financial support, collective action, social cohesion), social influence (e.g., subjective social standing, control over business earnings), and social norms (perceived norms around women’s economic activity). Self-oriented personal factors included measures of beliefs about the self (self-efficacy), one’s future (optimistic future expectations), and emotion regulation (mental health).^*^

This division of personal-relational factors has distinct limitations: Certainly, some variables classified as personal can entail relational factors and vice versa (e.g., high self-efficacy from advancing one’s group, good mental health from strong relationships). While this classification merits more precise tests, here it allows us to explore these broad dimensions and, in so doing, to expand upon the canonical Western literature on motivation, which has focused predominantly on personal self-regulatory factors (e.g., self-efficacy, optimism) while giving less attention to relational factors (14, 40, 42). An exploratory factor analysis largely supported this classification (*SI Appendix,* Table S1) and, notably, it follows the classification used in the original policy experiment (6).

## Results.

**Fig. 3.**
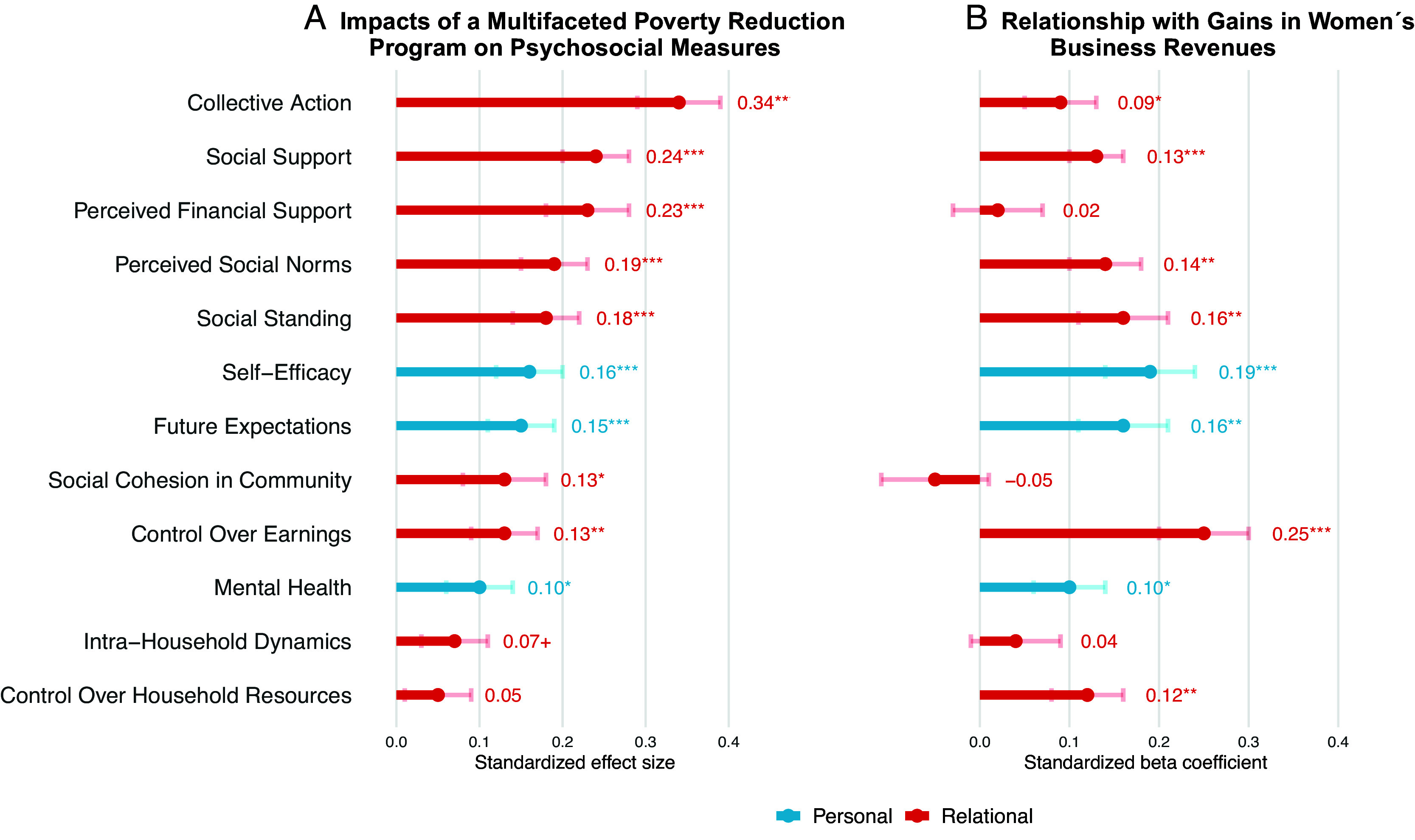
Impacts of the multifaceted poverty reduction program on personal and relational measures (*A*) and their relationships with women’s economic gains at the 6-mo follow-up (*B*) in the policy experiment. Panel *A* presents comparisons of the Psychosocial and Control arms [n = 2,208 for all indices, except control over household resources (n = 2,098)]. Panel *B* presents coefficients from separate regressions of women’s business revenues on each psychosocial factor in the Psychosocial arm, controlling for baseline revenue values [n = 1,056 for all indices, except controls household resources (n = 999)]. ^†^*P* < 0.10, *P < 0.05, **P < 0.01, ***P < 0.001. Error bars represent ±1 SE.

We find support for both relational and self-oriented personal factors as possible mechanisms of the economic gains produced by the program. Fig. 3 displays measures rank ordered by the standardized size of the causal impacts of the poverty reduction program (Psychosocial versus Control arm) and shows significant impacts on most psychosocial outcomes (Fig. 3*A*). Further, we statistically compared program impacts on relational and personal outcomes using a multivariate linear model: Overall the Psychosocial version of the program led to significant improvements on the composite of relational outcomes (*β* = 0.17 (95% CI = 0.14, 0.21), *t*(4417) = 7.91, *P* < 0.001) and on the composite of personal outcomes (*β* = 0.14 (95% CI = 0.08, 0.20), *t*(4417) = 4.37, *P* < 0.001). The size of these impacts did not significantly differ, given overlapping 95% CIs and according to a formal cluster-robust Wald test (*F*(1,135) = 1.41, *P* = 0.24).

Our primary level of theorizing and analysis is at the level of the composites. We do not draw confirmatory conclusions from significant impacts on individual measures as we did not predict specific patterns at this level; instead, we see them as indicating promising areas for future research, including for refining measurement and replicating effects. With that understanding, some of the largest impacts of the Psychosocial version of the program were on measures related to women’s social capital. Women were more likely to engage in “collective action” through community organizations and projects and to feel that they could rely on their community for “social support” and “financial support” in times of need. They also reported higher “social standing” in the community, specifically feeling more listened to and respected. They also perceived that their community viewed women’s economic activity as more common and acceptable (“social norms”). These findings suggest a strengthening of women’s relationships, social capital, and social position. In addition, women experienced significant improvements in self-oriented personal factors, including feeling greater “self-efficacy” to achieve their goals, more optimistic “future expectations,” and improved “mental health.” Notably, these relational factors and personal factors, as composites, were significantly correlated with each other in the Psychosocial arm, *r*(1054) = 0.38, *P* < 0.001 (see *SI Appendix*, Table S2 for intercorrelations among variables).

In turn, these positive relational and personal dynamics were significantly associated with women’s economic gains in the multifaceted program (Fig. 3*B*). We carried out a multivariate regression of women’s business revenues at the 6-mo follow-up on the composites of relational factors and personal factors, controlling for baseline revenues, within the Psychosocial treatment arm. The composite of relational factors significantly predicted women’s economic gains in the program [standardized *β* = 0.41 (95% CI = 0.23, 0.59), *t*(1052) = 4.51, *P* < 0.001], as did the composite of personal factors [standardized *β* = 0.18 (95% CI = 0.06, 0.30), *t*(1052) = 2.96, *P* = 0.003]. A Wald F-test indicated that the predictive power of the relational factors was marginally but not significantly greater than that of the personal factors (Δ*β*: 0.23, *F*(1,1052) = 3.32, *P =* 0.069).

While our theorizing focused on the predictive power of relational and personal factors in general, again it was informative to observe variability in the coefficients on individual measures. The factors that most strongly predicted women’s revenue gains related to their social influence in the household (“control over earnings”) and community (“social standing”), as well as to positive beliefs about their capabilities (“self-efficacy”) and their futures (“future expectations”).

This analysis showed that in this cultural context relational factors, such as subjective social standing, mattered to women’s economic advancement as much as hallmark self-oriented foundations of motivation, such as self-efficacy and optimism. Moreover, adding the composite of relational factors as predictors explained additional variance in women’s economic gains above and beyond personal factors and baseline revenues (*F*(1,1052) = 20.34, *P* < 0.001). These findings suggest the importance of relational processes, in addition to self-oriented ones, in the successful empowerment of women’s agency in this context. However, this study did not allow us to identify which types of psychosocial processes stemmed from which model of agency.

Most importantly, given the program’s dual-model intervention design, it leaves open the question: Were program features reflecting an interdependent model of agency critical to its success in reducing poverty or would features reflecting an independent model, requiring no cultural attunement, have been sufficient? Next, Study 3 isolates the causal effects of interdependent and independent models of agency on women’s economic gains in this context.

## Study 3: The Causal Economic Impacts of Emphasizing an Interdependent or Independent Model of Agency Over 1 y.

A preregistered field experiment, Study 3 separately tests the causal impacts of interventions highlighting an interdependent or an independent model of agency, compared to a control, on women’s economic outcomes over 1 y, as well as on a subset of personal and relational measures. As such, it evaluates whether the effectiveness of a psychosocial intervention depends on its degree of cultural fit. This experiment compares two supplemental intervention conditions to a control in which no supplemental intervention was delivered (n = 2,628). The brief, single-session interventions were designed to emphasize one of the two models of agency displayed in the film about Amina that had been included in the community event and life skills trainings in Study 2. The *relational agency* intervention emphasized interdependent agency—goal pursuit grounded in collective advancement and social harmony. The *personal agency* intervention emphasized independent agency—goal pursuit grounded in self-advancement and self-direction. This experiment was embedded within the Psychosocial and Full arms of the policy experiment in which all participants received the psychosocial interventions (Fig. 1). As such, we understand the interventions tested here as reinforcing one model of agency or the other (i.e., as supplemental). This experiment was randomized at the individual level with the women program participants. *SI Appendix,* Table S3 describes the sample and shows that balance was achieved on key variables across experimental conditions.^†^

We assess economic outcomes, as well as some psychosocial outcomes 1 y later in a 45-min follow-up endline survey. This abbreviated survey included a subset of measures from Study 2. Economic outcomes included food security, business engagement, and business performance. Relational measures assessed partner dynamics, household interpersonal dynamics, women’s control over their earnings, social standing, social support, and social cohesion. Personal measures assessed well-being, self-efficacy, and future expectations. Given the relatively limited number of variables for each category and to control the rate of false positives, primary analyses are conducted on economic, relational, and personal composite indices.

We preregistered the hypotheses that both interventions, given their focus on aspirations and goal pursuit, may enhance personal sources of motivation relative to the control condition; however, the relationally oriented intervention would also activate relational mechanisms important to women’s socioeconomic success and, ultimately, improve their economic outcomes over time, as compared to control. While we measured exploratory psychosocial variables (such as hypothetical scenarios) immediately postintervention among the treated groups and preregistered comparisons across interventions, budgetary and logistical constraints prevented data collection from the control group at that timepoint. We focus our analyses and discussion on the 1-y endline results given that this survey included data from the control group (our primary comparison group), assessed more standard economic and psychosocial measures (as used in Study 2), and reflected real-world impacts.

**Fig. 4.**
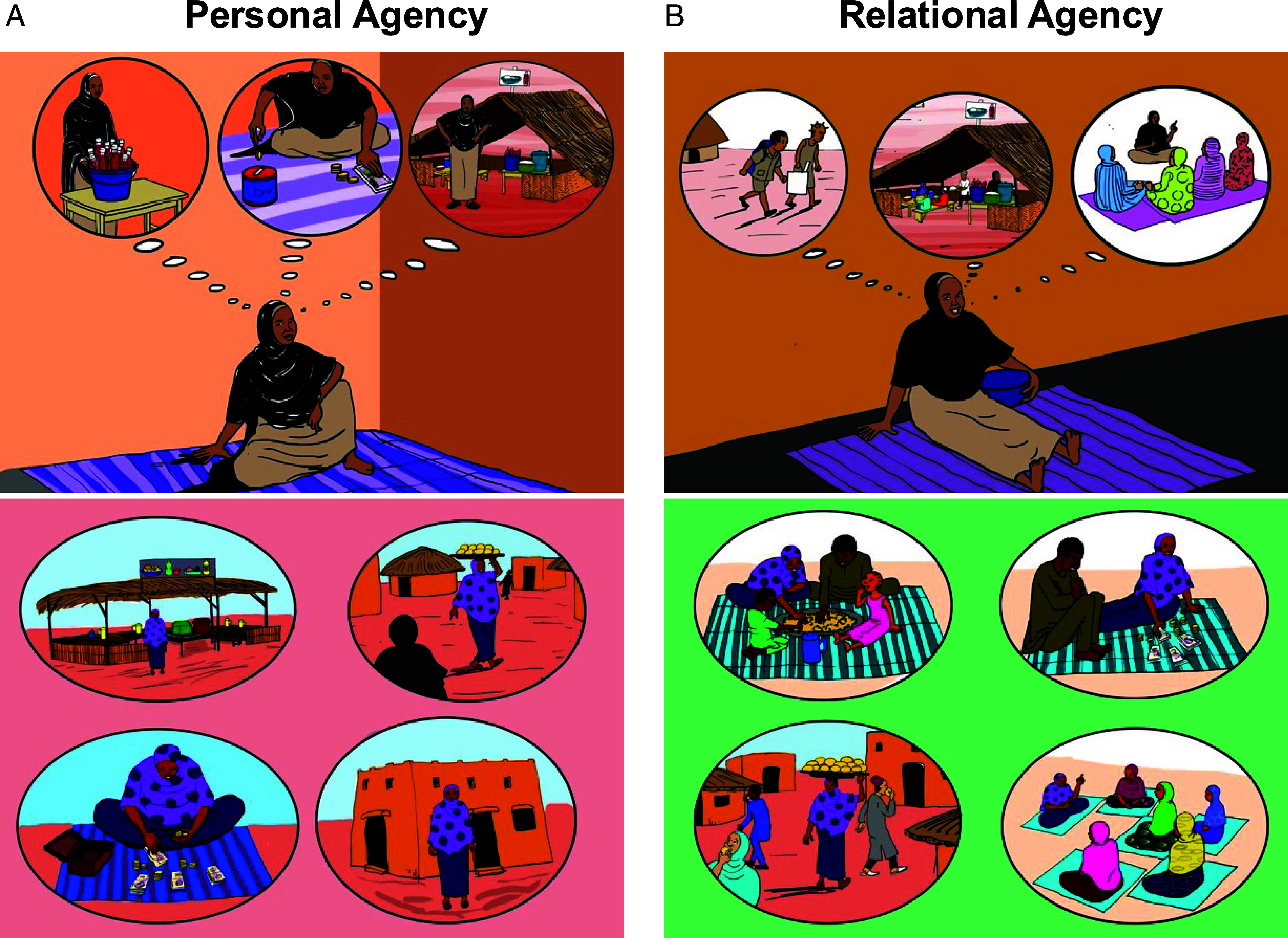
Sample images from the *personal agency* (*A*) and *relational agency* (*B*) intervention videos displayed in Study 3.

The brief psychological interventions comprised an approximately 4-min video recap of Amina’s story and 25-min guided reflection exercise. Fig. 4 shows sample images from the videos. Videos were matched on all characteristics where possible, including character depictions, features of the scenes, choice of business, and business trajectory. However, they differed in how they represented Amina’s motives and how she pursued her goals. In the *relational agency* condition, the recap portrayed Amina as becoming a well-regarded entrepreneur in her community by being respectful of elders, collaborative, and generous. In the *personal agency* condition, the video portrayed Amina as becoming a standout entrepreneur by being proactive, innovative, strategic, and competitive. Following the video, participants completed a guided exercise on goal setting and strategies. In the *relational agency* intervention, the exercise focused on guiding participants to set goals, articulate possible collective benefits from achieving those goals, anticipate possible barriers, and identify social strategies for overcoming barriers. It reflected features of interdependent agency, as identified in the formative qualitative and quantitative research reported in Study 1. Given this culturally grounded design, we consider this intervention to be more culturally wise. The *personal agency* intervention followed a similar format but was based on motivational exercises developed and tested primarily in Western populations, specifically mental contrasting and implementation intentions and personal initiative training (30–32, 38, 39). The exercise guided participants to set goals, articulate anticipated positive feelings from achieving those goals, anticipate possible barriers, and identify if-then strategies for overcoming those barriers.

In interpreting our results, we preregistered comparisons of each intervention to the control, rather than to each other. This is because comparisons across interventions would have required a prohibitively large sample size.^‡^ This prioritization aligns with evaluation sciences in other disciplines. The Food and Drug Administration, for instance, requires the evaluation of the effectiveness of new medical treatments against a control, not other standards of treatment. This practice again reflects the pragmatic constraints of testing the comparative effectiveness of treatments against each other (e.g., limitations of statistical power) (43). In the psychological and behavioral sciences as well, even large-scale field experiments and megastudies often find interventions to outperform controls but not each other (44, 45).

## Results.

**Fig. 5.**
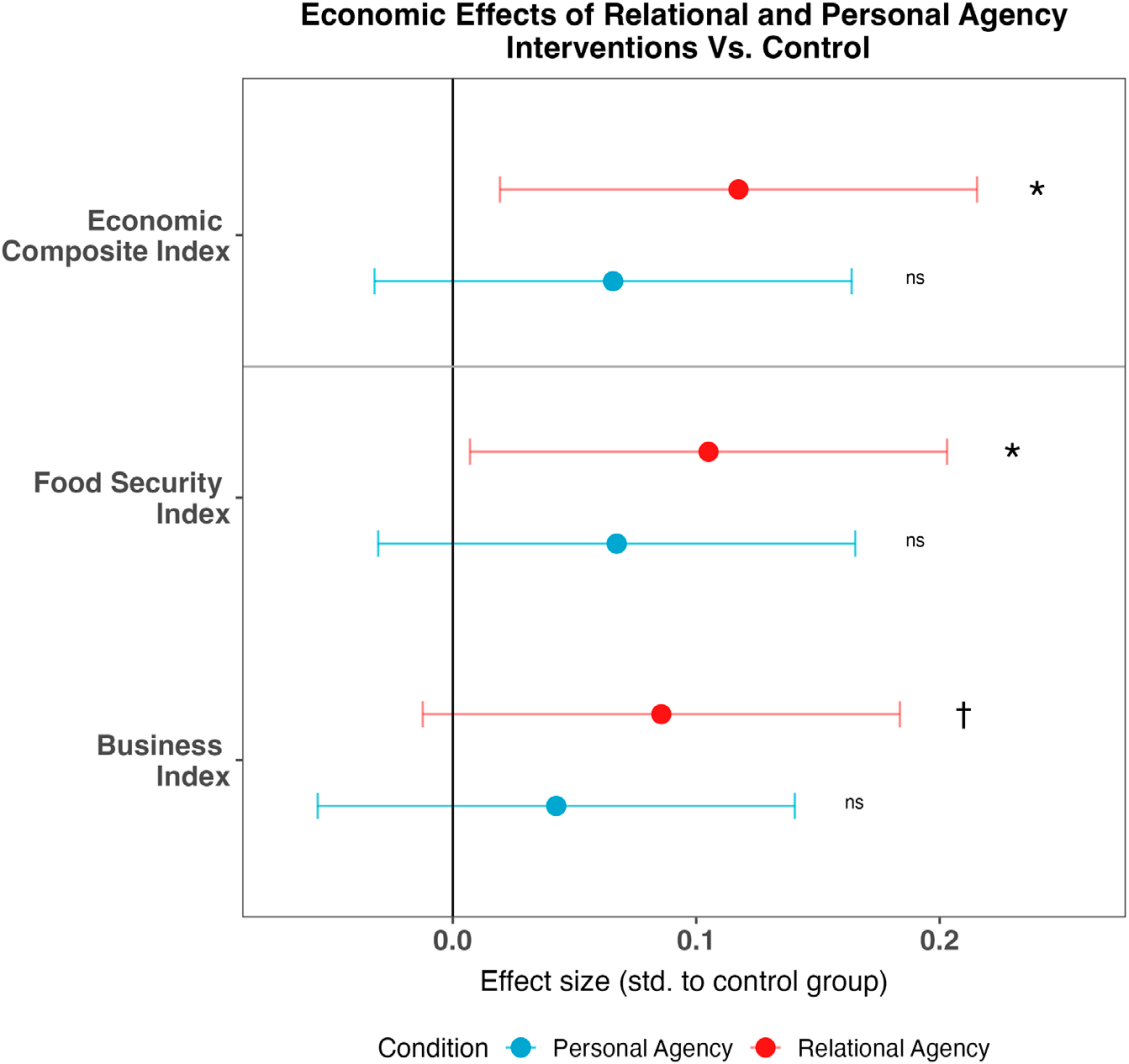
Impacts of relational agency and personal agency interventions on economic outcomes at the 1-y endline in Study 3. The economic composite index is a composite of the food security and business omnibus indices, which included a subset of variables from the policy experiment (Study 2). Point estimates represent the effect of each agency intervention condition compared to the control group in terms of SD (standardized to the control condition’s mean and SD). Regressions control for randomization strata, and SE are robust. Error bars are 95% CI. ^†^*P* < 0.10, *P < 0.05, *ns* indicates *P* > 0.10.

As displayed in Fig. 5, the *relational agency* intervention caused significant gains 1 y later on the primary index of economic outcomes compared to control (*d* = 0.12, SE = 0.05, *P* = 0.013), i.e., above and beyond those achieved by the multifaceted program in Study 2. This gain arose from increases in both subindices of food security and women’s business outcomes. As an illustration, women in the *relational agency* condition were less likely to report severe forms of food insecurity, according to the Food Insecurity Experience Scale. In the control group, 46% of women reported that their households had gone hungry or gone for an entire day without food due to insufficient resources in the past 12 mo. Yet, in the *relational agency* condition, this rate fell by 5.7 percentage points (*β* = −0.05, SE = 0.02, *P* = 0.025). Women in this condition were also marginally more likely to own or manage their own business compared to those in the control condition (*β* = 0.03, SE = 0.02, *P* = 0.079).

While directionally positive, the effect of the *personal agency* intervention was 40% smaller in magnitude and did not reach statistical significance on the overall economic composite (*d* = 0.07, SE = 0.05, *P* = 0.169), or on either subindex of food security or business engagement (see Fig. 5 for indices and *SI Appendix,* Table S5 for items within indices) [although the *personal agency* did reduce rates of severe food insecurity in particular (*β* = −0.05, SE = 0.02, *P* = 0.025)].

We consider the causal impacts of the agency interventions on women’s economic gains to be the primary test of our hypothesis. However, we also assessed psychosocial measures both as important outcomes on their own and as potential mechanisms of economic gains. Notably, as with the economic gains, any impacts on these measures would have to be observed on top of the gains already achieved by the program described in Study 2.

As predicted, both relational and personal agency interventions led to improvements on a composite of self-oriented personal measures at endline, *β* = 0.12, SE = 0.04, *P* = 0.010; *β* = 0.13, SE = 0.05, *P* = 0.004, respectively. Examining constituents of this composite (Table 1), both interventions led women to report more optimistic expectations for their socioeconomic mobility. Both also generated marginally or significant positive gains in subjective well-being and, for the *personal agency* intervention, in self-efficacy as well.

**Table 1.** Impacts of personal agency and relational agency interventions on personal and relational outcomes at 1-y endline in Study 3

		Personal agency	Relational agency
(1)	(2)	(3)	(4)
Measure	df	Coefficient (SE) Robust *P-*value Cluster robust *P-*value	Coefficient (SE) Robust *P-*value Cluster robust *P-*value
**Psychosocial composite index**	**2,487**	**0.10**	**0.12**
		**(0.05)**	**(0.05)**
		0.043*	0.015*
		**0.062^†^**	0.032*
**Personal composite index**	**2,487**	**0.13**	**0.12**
		**(0.05)**	**(0.04)**
		0.004**	0.010*
		0.011*	0.025*
Well-being	2,487	0.09	0.08
		(0.05)	(0.05)
		0.049*	0.089^†^
		0.069^†^	0.096^†^
Self-efficacy	2,473	0.09	0.02
		(0.05)	(0.05)
		0.068^†^	0.595
		0.093^†^	0.643
Future expectations	2,473	0.12	0.15
		(0.05)	(0.05)
		0.019*	0.002**
		0.058^†^	0.012*
**Relational composite index**	**2,487**	**0.05**	**0.09**
		**(0.05)**	**(0.05)**
		**0.338**	**0.075** **^†^**
		**0.344**	**0.128**
Partner dynamics	2,226	0.07	0.08
		(0.05)	(0.05)
		0.169	0.102
		0.181	0.157
Household interpersonal dynamics	2,487	0.03	0.10
		(0.05)	(0.04)
		0.458	0.021*
		0.507	0.045*
Control over earnings	2,473	−0.02	−0.03
		(0.05)	(0.05)
		0.627	0.584
		0.578	0.613
Social standing	2,473	0.03 (0.05)	0.05
		(0.05)	(0.05)
		0.527	0.318
		0.582	0.394
Social support	2,487	0.09	0.09
		(0.05)	(0.05)
		0.057^†^	0.077^†^
		0.114	0.125
Social cohesion	2,473	−0.05	0.00
		(0.05)	(0.05)
		0.300	0.982
		0.361	0.985

Note: Columns 3 and 4 are obtained from regressions comparing the *personal agency* and *relational agency* conditions, respectively, to the control condition, controlling for randomization strata. Point estimates represent the effect of each psychosocial intervention condition compared to control in terms of SD (standardized to the control condition’s mean and SD). ^†^*P* < 0.10, ^*^P < 0.05, ^**^P < 0.01, ^***^P < 0.001.

On the composite of relational measures, only the *relational agency* intervention produced marginally significant improvements compared to control (relational agency: *β* = 0.09, SE = 0.05, *P* = 0.075; personal agency: *β* = 0.05, SE = 0.05, *P* = 0.338). In the *relational agency* condition, women reported improved interpersonal dynamics within their households, specifically feeling closer to members of their households on a visual measure of interpersonal closeness versus distance (46) (*β* = 0.11, SE = 0.04, *P* = 0.008) and, marginally, feeling more well-regarded by their household for their economic activities (*β* = 0.09, SE = 0.05, *P* = 0.070). No significant effects were observed on other relational outcomes for either agency intervention, including social standing, women’s control over earnings, partner dynamics, and social cohesion, with the exception of marginal effects on social support (Table 1). The finding that the *relational agency* intervention affected self-oriented personal measures in addition to relational measures is noteworthy and consistent with our theorizing of personal and relational processes as interconnected mechanisms of effective interdependent agency in this context.

Interestingly, these improvements in household interpersonal dynamics paralleled observed gains in *household* business outcomes, according to a post hoc exploratory analysis. For a random subsample of households participating in Study 3 who were also surveyed in Study 2’s policy experiment (approximately one-fifth, n = 457), we were able to collect data on “household-owned businesses,” i.e., off-farm businesses owned and managed by other members of the household (e.g., husbands, cowives, in-laws). As displayed in *SI Appendix,* Table S7, in households in which the woman program participant had been randomized to the *relational agency* condition, businesses run by other household members showed greater outcomes at the 1-y follow-up, according to a composite of business engagement and performance, compared to the control condition. For instance, while households in the control condition reported monthly total business revenues of approximately US$70 PPP, households in the *relational agency* intervention saw revenues of US$111 PPP, a 59% gain. While directionally positive, no significant effects were observed among households assigned to the *personal agency* condition, in which revenues amounted to US$83 PPP.^§^

Why might household interpersonal dynamics have improved and household-owned businesses grown in the *relational agency* condition? As part of the guided exercise in this condition, we invited women to think through how they could effectively navigate conflict in the household and maintain harmony while pursuing their business goals, in the form of giving advice to another woman. One possibility is that the *relational agency* intervention better equipped women to engage in negotiated agency (24) and build shared aspirations with their husbands and other family members. For example, one respondent advised other women “to explain the advantages of business development for the education of their children and the well-being of their household.”

While not preregistered or our primary focus for reasons elaborated above, we ran post hoc comparative analyses of interventions at endline, and we found that differences between the relational and personal agency interventions did not reach statistical significance on the economic composite index (difference of *d* = 0.05, SE = 0.05, *P* = 0.343), personal composite index (difference of *d* = −0.02, SE = 0.05, *P* = 0.693), or relational composite index (difference of *d* = 0.04, SE = 0.06, *P* = 0.446) (Table 1 and *SI Appendix*, Table S5).

We did, however, expect and preregister intervention differences on exploratory mechanism variables collected immediately postintervention, given the lab-in-the-field design, but found none (*SI Appendix,* Table S8) with one exception: Women in the *relational agency* condition were marginally less likely than women in the *personal agency* condition to express reputational concerns, specifically to anticipate being seen negatively by family and community while pursuing their economic activities (*β* = −0.03, *P* = 0.088), supporting one of three preregistered hypotheses. Unfortunately, without data from the control group at this timepoint, we cannot infer whether the null results mean that the interventions had no effects or similarly positive effects. This postintervention survey was also subject to potential methodological concerns (e.g., nonvalidated measures; social desirability processes as measures were assessed by the intervention implementer), warranting a prioritization of endline results.

Overall, while both agency interventions set in motion self-oriented personal processes (e.g., greater optimism about one’s future), only the *relational agency* intervention affected some relational processes (e.g., feeling closer to others in the household) and, most importantly, translated into significant economic gains, including greater food security. These multidimensional impacts of the *relational agency* intervention are striking for several reasons: They resulted from a single 30-min session, were detected on meaningful economic outcomes 1 y later, and were achieved on top of the gains produced by the more intensive multifaceted program in Study 2. These gains imply the fundamental importance of emphasizing a culturally rooted conceptualization of agency.

## Discussion

Amartya Sen argued, “Freedoms are not only the primary ends of development, they are also among its principal means” (47). Agency is one such central freedom. Yet *how* people enact agency varies according to the sociocultural and economic contexts they seek to navigate. To support people’s agency, our findings suggest that programs are most likely to be effective when they are contextualized to the model of agency predominant in the local cultural context—that is, when they are culturally wise.

Three studies revealed the centrality of interdependence to women’s agency in rural Niger. They also suggested possible psychosocial mechanisms through which this agency can translate into upward economic trajectories. Study 1 showed an interdependent model of agency grounded in social respect, social harmony, and collective benefits to be predominant in this context, being broadly valued, practiced, and viewed as important to women’s economic success. This contrasted with assumptions of a Western sample, who centered an independent model grounded in self-initiative and future orientation. Study 2 provided evidence for the importance of both relational and personal psychosocial mechanisms of a highly effective multifaceted poverty reduction program. Notably, relational factors, such as perceived social standing and support, accompanied and predicted women’s economic gains in addition to and as much as personal factors like self-efficacy, the latter of which have been the focus of canonical Western motivation research.

Critically, Study 3 provided causal tests of interventions grounded in independent and interdependent models of agency compared to a control. It found that a “culturally wise” *relational agency* intervention—which emphasized collective benefits, social harmony, and respect in the pursuit of economic development (based on Study 1)—significantly improved women’s economic outcomes and activated personal and some relational psychosocial processes, above and beyond the multifaceted program tested in Study 2. These effects were not statistically greater than those of the *personal agency* intervention reflecting an independent model. However, the latter—which was derived from prototypical, evidence-supported Western motivational interventions—achieved limited effects compared to control, significantly shifting only personal factors but neither economic nor relational outcomes. Combined with Study 2, these patterns are consistent with a model of interdependent agency in which personal goal pursuit will be most successful and afforded in this cultural context when seen as enacted for interdependent motivations and in ways that strengthen one’s relationships.

As the psychological and behavioral sciences globalize and enter policy circles, our results underscore the need for theory and measurement to expand beyond the independent individual, and associated self-oriented motivational forces, and to also capture the range of relational forces that animate interdependent actors (21). Notably, had we relied exclusively on Western motivational defaults grounded in independence and factors like self-initiative and future orientation (Study 1), we would have overlooked the importance of interdependence and of relational processes in women’s economic advancement (Studies 1-2) and failed to identify an effective agency intervention (Study 3). Notably, the positive impacts of the *relational agency* intervention on women’s economic advancement (Study 3) reveal the tangible benefits of broadening theories of motivation and human behavior (40).

From a policy perspective, past studies have documented the multidimensionality of poverty, including its psychosocial features (48, 49). Here, we show *how* poverty reduction programs may begin to address these psychosocial factors across diverse cultural settings. This set of studies provides a methodological blueprint for identifying predominant models of agency in a given context, for assessing related psychosocial factors, and for embedding those models in intervention designs.

Several limitations are important to examine in future research. Study 3 did not find and was not powered to detect differences across the two interventions. In addition, it was conducted in just one cultural context. Comparative experiments across global regions could clarify the role of culture and context in shaping responsiveness to different interventions (50). For instance, future research could design agency interventions to reflect models of independence and interdependence, such as self-enhancing independence in the West, self-promotive interdependence in sub-Saharan Africa, or self-effacing interdependence in East Asia (25), and, second, assess their relative effectiveness within one culture compared to another.

Different iterations of agency interventions could also be tested to meet sources of variation within a country. For instance, a “personal initiative” intervention teaching personal proactivity, future orientation, and competitiveness has been found effective over time among urban male entrepreneurs in Togo but not female entrepreneurs (51). A promising direction for improving agency interventions for women in West Africa could focus on women’s social standing and control over earnings in the household—both typically lower than men’s and, in our studies, highly predictive of business revenues (Study 2) yet not further improved by the single-session agency interventions (Study 3), beyond the impacts of the multifaceted program (Study 2).

To build greater interpretive power around foundations of women’s agency across cultures (37), future research could use experimental tasks and qualitative methods to more deeply investigate the cultural meaning of key psychosocial constructs (such as peacefulness and social cohesion) and unpack how relational and personal factors may interact in local models of agency (52). In addition, given the suggestive but not conclusive effects of the *relational agency* intervention on indicators of improved interpersonal dynamics (e.g., greater interpersonal closeness, fewer reputational concerns), future research should seek to replicate these effects and, more generally, to study a wider array of relational processes [e.g., communication behaviors (53), reputation management (54)].

We are optimistic that the behavioral and psychological sciences can help solve complex global problems. However, our findings suggest that this potential may rest, in part, on the sensitivity of scholars and policymakers to the world’s cultural diversity. With research at the intersection of cultural and social psychology, behavioral science, and development economics, we hope that interdisciplinary and cross-cultural teams can attune interventions to cultural context and generate more innovative solutions for tackling global poverty.

## Materials and Methods

### Study 1.

Study 1 was not preregistered as these measures were exploratory and descriptive in nature.

#### Participants.

Data for Niger participants were collected through the baseline survey of the policy experiment (n = 4,717) (see Study 2 for details) or otherwise from respondents assigned to the control group in Study 3 at the 1-y follow-up (n = 1,216) (see Study 3 for details). For the US sample, we recruited 302 respondents based in the United States from CloudResearch’s MTurk Toolkit platform to take a descriptive survey. The sample was on average middle income, with the modal annual household income being $40,001-$60,000; 57.6% identified as male, 41.7% as female, and 0.7% self-described; 42.4% had a 4-y college degree and 20.9% had some college; 70.9% identified as White, 12.9% as Black, 5.3% as Asian, and 4.6% as Hispanic. On a seven-point scale of political ideology (1 = Strongly liberal to 7 = Strongly conservative), the sample scored on average 3.14 (SD = 1.78).

#### Data collection.

See *SI Appendix*, section S1 as well as Study 2 for measures.

### Study 2.

Study details, including on sample, randomization strategy, treatments, outcomes, and analysis, is reported in full in Bossuroy et al. (6). The research protocol was preregistered in the AEA RCT registry: https://www.socialscienceregistry.org/trials/2544.

#### Participants.

Participants were women from low-income households in predominantly rural areas of Niger who were eligible for participation in the multifaceted program versions of the Sahel Adaptive Social Protection (ASP) program on the basis of a Proxy Means Targeting score and other methods seeking to identify poor and vulnerable households. Eighty-five percent of these households were found to fall below Niger’s national poverty line (6). The selection process is detailed here: http://documents.worldbank.org/curated/en/387791524060631076/pdf/WPS8412.pdf.

#### Interventions.

See *SI Appendix*, section S1 for descriptions of all treatment arms and details on the psychosocial interventions.

#### Procedure.

See *SI Appendix*, section S1 for a description of measures.

### Study 3.

The research protocol was preregistered in the AEA RCT registry https://www.socialscienceregistry.org/trials/3570. Details on the wording and construction of measures can be found there as well.

#### Participants.

The study includes 2,628 women in 33 villages across six communes (see *SI Appendix,* Fig. S1 for a map of the commune locations). Study respondents were recruited from the pool of program participants in villages assigned to the Psychosocial and Full arms of the policy experiment (i.e., those program versions that included psychosocial interventions) (see Study 2). Women in this sample were on average 34 y old (SD = 14.11) and had 0.64 y of education (SD = 1.97). The average household size was 9.54 members (SD = 4.64). Most participants were not heads of their household (88%) and were not nomadic (89%). The average distance to the nearest market from each village was 73 min (SE = 3.52) and to the nearest water source was 12 min (SE = 1.14). Among those who participated in the endline survey, a minority (16%) of women respondents owned a personal cellphone. While religious identification was not asked, a vast majority of Nigeriens practice Islam.

#### Randomization strategy.

We randomized 1,332 participants to one of two brief psychosocial motivational interventions (“personal agency” or “relational agency,” n = 666 per group) and 1,296 to a control condition. These randomizations were stratified by the timing of the delivery of the multifaceted program described in Study 2 (Early: February–March/Late: April), program version (Full or Psychosocial), and participation in the policy experiment (Yes/No). Randomization occurred at the individual level. Women participants in the multifaceted program were also organized into groups for other program activities (e.g., skills trainings). On top of the individual-level randomization, we included a group-level randomization, such that those groups were assigned to have 25% (n = 36 groups), 50% (n = 36 groups), 75% (n = 36 groups) of their members treated with either psychosocial intervention. These groups had between 11 to 33 women participants, with an average of 25.

Randomization produced well-balanced conditions on preregistered key sociodemographic variables, including a poverty proxy score, age, nomad status, living in a hamlet outside the village versus inside the village, and household head status, as well as randomization strata (*SI Appendix,* Table S3). Note that randomization was not balanced on the randomization stratum of participation in the policy experiment trial due to error; however, women had initially been randomly selected within villages into participation in that trial. Also, all analyses control for this variable, as with other randomization strata.

Response rates in the endline survey were high overall, with 95% of the sample participating. Attrition was minimal in magnitude with 5% lost to follow-up (n *=* 135) (*SI Appendix*, Fig. S2). However, attrition was marginally differential across conditions: 5.7% (n = 74) in the control, 6.0% (n = 40) in *personal agency*, and 3.2% (n = 21) in *relational agency*. According to a logistic regression controlling for stratification variables and applying robust SE, those in the *relational agency* condition were less likely to show attrition than those in the control condition (*P* = 0.038) but attrition did not differ between the control and *personal agency* conditions (*P* = 0.446). Taking all observable covariates as a set, we found no evidence of overall differential attrition according to a joint hypothesis test on the interactions between covariates and condition assignment in a regression predicting attrition (*F*(16, 2601) = 0.984, *P* = 0.471). However, regressing attrition on each covariate separately, attrition was found to be associated with older age (*β* = 0.01, *P* = 0.025) and with status as head of household (*β* = 0.50, *P* = 0.030). *SI Appendix,* Table S4 presents attrition analyses. *SI Appendix,* Table S6 presents robustness analyses which show that patterns of results on economic and psychosocial outcomes remain the same when controlling for these two variables of age and status as head of household.

#### Statistical power.

The study was powered for a minimum detectable effect (MDE) size between each of the psychosocial interventions and the control condition of Cohen’s *d* of 0.14 and the psychosocial interventions (pooled) and the control condition of Cohen’s *d* of 0.11, with 80% power using a two-tailed independent samples *t* test at α = 0.05. This target MDE required n = 613 for each of the two psychosocial intervention arms and 1,192 for the control arm. Assuming a rate of 8% for attrition, our target sample size was n = 2,628 (n = 1,296 for the control condition and n = 666 for each of the treatment conditions). See *SI Appendix*, section S1 for further rationale and empirical benchmarks for power analyses for both endline and postintervention analyses.

#### Interventions.

This embedded experiment was conducted after the community sensitization which was designed to introduce villages to the multifaceted poverty reduction program and which consisted of a 20-min film and community discussion (see Study 2 for details). That film depicted the story of a role model named Amina. In this embedded experiment, participants were then randomized to a control condition or one of two psychosocial interventions that reinforced different interpretations of behavior modeled in that film. In the *personal agency* condition, the video portrayed Amina as becoming a standout entrepreneur by being proactive in planning her business goals, innovative in her choice of products, and strategic and competitive in the marketplace. In the *relational agency* condition, the recap portrayed Amina as becoming a respected entrepreneur in her community by actively seeking counsel from elders on developing her businesses, by collaborating with her husband in decision-making, and by being generous with other women in her community through sharing her knowledge.

Then, participants completed a 25-min guided exercise. In the *personal agency* condition, a mental contrasting and implementation intentions exercise (30–32) prompted participants to set goals, imagine possible positive feelings that could result, identify possible barriers, and create if-then strategies to overcome those barriers. In the *relational agency* condition, participants completed a motivational goal-setting and planning exercise in a similar format but this exercise focused on relational goals, barriers, and social strategies. See *SI Appendix*, section S1 for the content of the videos and guided exercises in each intervention.

#### Procedure.

During these individual-level sessions, participants were guided by female field staff through one of the two psychosocial interventions in the participant’s desired language (Hausa or Zarma). The video was displayed on tablets followed by a series of prompts asked aloud by field staff, which together lasted approximately 30 min. Field staff recorded summaries of participants’ qualitative responses. Field staff then led participants through a series of psychosocial outcome measures, including hypothetical economic scenarios designed to be relevant to the local population. These sessions lasted approximately 80 min in total and took place in a private space in or near the participant’s home. Field staff were blind to hypotheses and were trained by the survey firm and research teams.

An endline survey was conducted approximately 1 y later among the full sample. Female enumerators were blind to condition assignment and administered the survey to female respondents in a private space in or near their homes. Enumerators asked respondents a series of economic, psychological, social, and program-related outcome measures in the participant’s desired language.

See *SI Appendix*, section S1 for a detailed description of measures.

#### Empirical strategy.

Deviations from this plan are described in *SI Appendix*, section S3, and primarily reflect changes to align analyses with the policy experiment, as published in Bossuroy et al. (6).

To assess treatment effects at endline, our primary model compares each psychosocial intervention to a control condition, controlling for stratification variables used in randomization. We present robust *P-*values as primary but also display cluster robust *P-*values. Clustered SE were initially considered to account for the fact that women were organized into groups as part of the program and thus their outcomes are likely to be correlated and because a group-level randomization was conducted on top of the individual-level randomization. However, recent evidence (55) suggests that cluster robust *P*-values may be overly conservative for this individual-level randomization design. In addition, although we preregistered directional improvements and one-sided tests from the interventions, we present more conservative two-sided tests.

## Supplementary Material

Appendix 01 (PDF)

## Data Availability

Data from the policy experiment used in Studies 1 and 2 are available at https://microdata.worldbank.org/index.php/catalog/4294 (56). Data from the embedded experiment used in Studies 1 and 3, and ancillary US data for Study 1, are available at https://microdata.worldbank.org/index.php/catalog/7859 (57). The reproducibility package, including R code for analyses, is available at https://reproducibility.worldbank.org/catalog/358 (58). The policy experiment (Studies 1 and 2) received approval from the Innovations for Poverty Action Institutional Review Board (#00006083). The embedded experiment (Studies 1 and 3) and ancillary US predictions (Study 1) received approval from Stanford University Institutional Review Board (#44074). All participants provided informed consent.

## Footnotes

^*^We analyze all psychosocial variables measured in Bossuroy et al. (6) except violence perceptions, which was considered a separate outcome in that study.

^†^We also randomized the proportion of individuals within a village (25%, 50%, or 75%) that were treated with one of the two interventions. *SI Appendix*, Tables S9 and S10 show that this village-level randomization of saturation had minimal to no effect on psychosocial and economic outcomes.

^‡^For example, to detect the observed difference across intervention conditions of 0.05 SD on our primary outcome, the economic composite, with 80% power would require a sample size of approximately n = 12,560 (6,280 per condition). See *Materials and Methods* section for power calculation assumptions and benchmarks.

^§^Monetary amounts are in PPP-adjusted USD terms, set at 2016 prices and deflated using Niger’s CPI published by the World Bank. In 2016, 1 USD = 242.553 XOF PPP.
