# Supplementary material for: How culturally wise psychological interventions can help reduce poverty
Source: Proc Natl Acad Sci U S A. 2025 Nov 13;122(46):e2505694122. doi: 10.1073/pnas.2505694122 (PMC12646240; doi:10.1073/pnas.2505694122)
Supplement: Supplementary file 1 — Appendix 01 (PDF) [file pnas.2505694122.sapp.pdf]

PNAS

Supporting Information for
How Culturally Wise Psychological Interventions Can Help Reduce Poverty

Catherine C. Thomas[abc]*, Patrick Premand[d], Thomas Bossuroy[e], Soumaila Abdoulaye Sambo[f], Hazel Rose Markus[g], Gregory M. Walton[c]

[a] Department of Psychology, University of Michigan, Ann Arbor, MI, 48109 USA
[b] Organizational Studies, University of Michigan, Ann Arbor, MI, 48109 USA
[c] Department of Psychology, Stanford University, Palo Alto, CA, 94305 USA
[d] Development Impact, Department Economics, World Bank, Washington DC, 20433 USA
[e] Social Protection and Labor, World Bank, Washington DC, 20433 USA
[f] Groupe de Recherche, d'Etudes et d'Action pour le Développement (GREAD), Niamey, Niger 10380
[g] Department of Psychology, Stanford SPARQ, Stanford, CA 94305

* To whom correspondence may be addressed: Catherine C. Thomas
Email: thomascc@umich.edu

This PDF file includes:

Supporting text
Section S1. Extended Methods
Section S2. Additional Analyses
Section S3. Pre-registration Deviations
Figures S1 to S2
Tables S1 to S10
SI References

Supporting Information Text

Section S1. Extended Methods

Study 1.

Measures. In the Niger sample, for the mental models question participants were asked "Of these 4 qualities, which is the most necessary for the success of a woman in her economic activities?" and were asked to rank order four options: working hard, having peace, showing independent initiative and being strategic, and having the correct social connections. They were then asked, "Of these 4 reasons, what is the main reason why women are not successful in their economic activities?" and were asked to rank order four options: not respecting others, not persevering in the face of obstacles, having tension in the household, and not having a personal plan for the future. These measures, as with all measures in Studies 1-3, were translated into Zarma and Hausa through a process of translation, backtranslation, and reconciliation to resolve discrepancies.

In the survey with the U.S. sample, we explained to participants that "The following questions ask for your predictions about the economic success of low-income women in the context of Niger." They were asked, "Of these 4 qualities, which do you predict will be the most important for women's economic success in this context?" and were asked to rank order four options: working hard, having peacefulness, showing personal initiative and being strategic, and having good social connections. They were also asked "Of these 4 reasons, which do you predict will be the most important for why women are not economically successful in this context?" and were asked to rank order four options: not respecting others, not persevering in the face of obstacles, having tension in the household, and not having a plan for the future. There were very slight alterations in the wording of these questions across samples for translation reasons. They were also asked to make a few other predictions. Sociodemographic information was collected at the end of the survey.

Study 2.

Interventions. See Bossuroy et al. (2022), including the Supplementary Information, for a full description of treatments. A summary of details most relevant to these secondary analyses is provided below.

The multi-faceted program versions of the Sahel Adaptive Social Protection program were targeted to women in low-income households to encourage and support them in expanding and diversifying micro-enterprises, which is a primary pathway for reducing poverty and food insecurity in this highly climate-affected region. This four-arm policy experiment included a control arm, which was a national safety net program providing monthly cash transfers of US$38.95 (in 2016 PPP). All three treatment arms included a core set of economic components, including the monthly cash transfers plus savings group formation, micro-entrepreneurship training, group coaching and market access facilitation. To those components, the Psychosocial arm added two psychosocial interventions, a community sensitization and a 1-week life skills training. The Capital arm added, instead, a large lump sum cash grant of US$311 (in 2016 PPP). The Full arm included all components.

The two psychosocial interventions were grounded in both interdependent and independent agency. The first psychosocial intervention—the community sensitization—was designed to introduce participating communities to the program. Both the film and group discussion prompted a construal of women's micro-entrepreneurship, a target outcome of the program, as a way to advance local values of social harmony, respect, and generosity. The 20-minute film modeled the story of a woman named Amina who became a successful entrepreneur through her initiative—both her exercising self-direction and planning and through reaching out to her family and peers for support and solidarity. Along the way, she overcame economic and interpersonal challenges. In the end, she shows self-confidence and shares her learnings with other women in her village. She also uses her own economic success to support her children's schooling and her husband's business. A subsequent moderated discussion prompted members of the community to articulate how Amina's story, and women's micro-entrepreneurship more broadly, may align with their goals and values. It also prompted the audience to set collective aspirations for the next generation and to identify coordinated behaviors that would advance those aspirations (55). This event was conducted with the approval of the village chief. Village elders, religious leaders, and

economic leaders were also invited as community members who would likely have influence over women's success in becoming micro-entrepreneurs. Program beneficiaries were also encouraged to invite their husbands, family members, and friends, i.e., people who could provide them instrumental and socioemotional support.

The second psychosocial intervention—1-week life skills training—was conducted in groups of approximately 20 women who came together to learn skills like goal setting and planning, decision-making, problem-solving, interpersonal communication, and leadership. In addition to teaching practical skills, these training sessions were intended to build women's sense of self-worth, confidence, and persistence. Women were also prompted to identify their values and their strengths as well as discuss their roles in and contributions to their families and communities. The same film from the community sensitization was used in several training sessions to role model the exercise of different life skills and link these skills to community values and contributions. The pedagogy of these trainings was grounded in participatory, problem-centered, personalized learning.

According to administrative data, attendance rates of beneficiaries at the community sensitization events was 89.3%. Attendance rates of beneficiaries in the life skills trainings was 93.8%, and 85% completed at least five of six sessions offered. According to administrative data collected in a subset of villages, the entire sensitization lasted approximately 1.5 hours on average, approximately 250 people attended the sensitization per village, and village chiefs and imams were consistently present. Approximately 18% - 30% of beneficiaries in attendance saw the film with their husbands and 30% with their children. Approximately 87% of beneficiaries in the psychosocial arms reported remembering the film's protagonist Amina in the follow-up survey.

Measures. See Bossuroy et al. (2022) Supplementary Information for a full description of measures. A summary of details most relevant to these secondary analyses is provided below.

Economic outcomes. Women's business revenues are computed from questions about revenue generated in the last month in which a business was operational. We winsorize revenues at the business-level at the 98th percentile. To get yearly amounts, we multiply this monthly revenue by the number of months a business was in operation in the last 12 months. To get the beneficiary's share, we divide revenues by the number of co-owners. Finally, we sum revenues across all beneficiary-owned/managed businesses.

Personal psychosocial outcomes. Mental health (17 items, α = .82) included CESD-R-10, a depression screener designed for community samples; functional disability items from SRQ-20; life satisfaction using an adapted Cantril ladder; sense of inner peace; and self-assessed mental health. Disability items, assessing somatic symptoms and role functioning, and self-assessed mental health item were included to capture cultural differences in mental illness symptoms (e.g., somatization) and functional impairments (1). Inner peace was considered a culturally specific indicator of well-being, according to qualitative piloting and West African studies (2). *Self-efficacy* (8 items, α = .76) captured judgments of one's capabilities, specifically in relation to problem solving, goal pursuit, and coping, and a related self-esteem question. Self-efficacy has been found to be a motivational keystone in Western settings, particularly of goal setting and pursuit (3). *Future expectations* (3 items, α = .76) gauged expectations for personal and intergenerational socioeconomic status as well as life satisfaction in the future, through adapted MacArthur ladders.

Relational psychosocial outcomes. The relational psychosocial outcomes were seen as potential determinants of women's economic outcomes in the low literacy, low resource, and normatively tight study setting where women's opportunities often come through their relationships and where reciprocal networks of support can be critical to cope with shocks. *Financial support* (3 items, α = .26) and *social support* (6 items, α = .66) assessed women's level of social capital. Financial support assessed the extent of one's financial support network, based on perceived ability to receive financial help in times of need and number of financial supporters. Social support assessed the extent of one's instrumental support network, based on the number of relationships one has for acquiring information, advice, and opportunities. Together, these questions capture whether beneficiaries are able to develop informal systems of support for economic resilience and opportunity.

Social standing (4 items, α = .75) assessed the MacArthur socioeconomic status ladder (4) and three context-specific ladders of community standing: being respected, having one's opinion followed, and showing moral behavior.

Social norms (8 items, α =.55) assessed descriptive and prescriptive norms supportive of women's economic engagement. The *descriptive norms* sub-index (4 items, α = .55) assessed perceptions of other women in the village engaging in economic activities, such as starting new activities, becoming vendors, and traveling outside the village. The *prescriptive norms* sub-index (4 items, α = .61) assessed perceptions of other men and women believing that women *should* engage in, i.e., social approval versus censure of, such activities.

Social cohesion and community closeness (9 items, α = .47) assessed social interdependence and collectivism, expectations of social support, feelings of trust and closeness, experienced tension (reversed), and number of enemies (reversed). Interdependence and collectivism items measure how individuals view themselves in relation to others, i.e., as similar, connected, and responsive versus separate, unique, and autonomous. Additionally, the number of enemies, or people who wish to sabotage your success, was included as a locally relevant indicator of social cohesion (5), particularly given that enemies can arise from envy and resentment following inequalities in new economic opportunities or resources.

Collective action (5 items, α = .34) assessed community engagement and support through the number of groups belonged to, the number of leadership positions held, monetary and volunteer contributions to community projects, and self-reported collective initiative. This measure assessed women's engagement in community leadership as well as the potential of the interventions to create indirect benefits to communities.

Intra-household dynamics (6 items, α = .33) assessed perceived quality of intra-household relationships with one's partner (3 items, α = .09) and one's household (3 items, α = .25). Items included feelings of trust, closeness, and support as well as experienced conflicts (reversed). Given that many economic and behavioral decisions are made at the household level in the study setting, this measure gauged the extent to which beneficiaries felt aligned with and supported by members of their household in decision-making or experienced tensions (reversed). It also assessed the Inclusion of Other in Self visual scale and sense of trust as indicators of intra-household closeness and positive relationality (6).

Of note, the indices of financial support and control over earnings were slightly modified from Bossuroy et al. (2022) to exclude objective economic variables.

Study 3.

Statistical power (continued). Our study design prioritized the comparison of interventions to control, and not between intervention comparisons, at endline for several reasons. For a benchmark, Lund et al. (2024) conducted a meta-analysis of psychological interventions to treat common mental disorders like depression on economic outcomes over time in low- and middle-income countries and found that they produce a meta-analytic effect, compared to control groups, of 0.16 standard deviations (7). This is comparable to the statistical power afforded by our design for comparisons of each intervention versus control—80% power for an MDE of Cohen's *d* of 0.14. We expected intervention-intervention differences to be smaller than intervention-control differences at endline because both agency interventions showcased positive role models and the possibility of better futures and, more generally, were based on promising evidence from other contexts; this would suggest directionally positive effects for both interventions and thus smaller differences across them. Consistent with this reasoning, large-scale field experiments and mega-studies of promising psychological interventions have found that they often outperform control groups on real-world outcomes but rarely each other (8, 9).

Our assumptions were different for outcomes measured immediately post-intervention among the treated. Notably, while we did not expect to be able to detect, nor pre-register, significant differences between interventions on real-world outcomes over time, we did expect, and pre-registered, some significant differences across intervention conditions on outcomes collected immediately post-intervention (but not in the control group). This is because this follow-up point constituted a lab-in-the-field design, and lab experiments can yield more precise, and potentially larger, effects than field experiment designs (10). *A priori* power analyses indicated that a sample size of n = 666 per condition would allow for an MDE of Cohen's *d* of 0.16 between interventions on outcomes collected immediately post-intervention, with 80% power using a two-tailed independent samples t-test at α = .05.

Interventions (continued). Below are the full details of the film scripts and guided exercises in the *personal agency* and *relational agency* interventions.

Key excerpts from introductory script for both interventions. “In the coming weeks, you will be starting several weeks of business and life skills trainings. We would like to tell you more and ask you questions about your participation in this program.” “A few weeks ago, you or others in your village saw a film about the story of a woman named Amina developing her activities. I'd like to show you a recap of this movie and then ask you some questions about how this story relates to your experiences.”

Personal Agency Intervention.

Introduction: The business and life skills training is intended to help you develop your activities and pursue your personal goals for your activities. The goal of the program is to increase the productivity and profits of beneficiaries' activities and to facilitate the development of new enterprises and steadier income. Through the business skills training and other measures, the program aims to help individuals advance their socioeconomic status, better cope with shocks in the future, and become more self-sufficient.

Film script by scene:

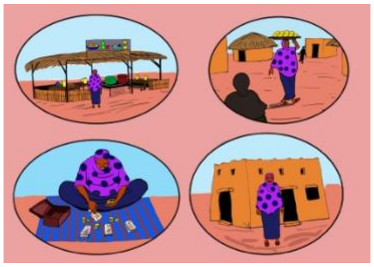 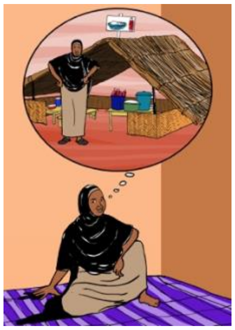	In the movie you saw, the woman envisioned a plan for growing her economic activities. She wanted to make changes so that she could have a better future. To do this, she started saving, learned a new activity, and reinvested in her activities. By pursuing new strategies, she was able to achieve her goals of advancing her financial status and financial security, and she was able to become a successful entrepreneur.
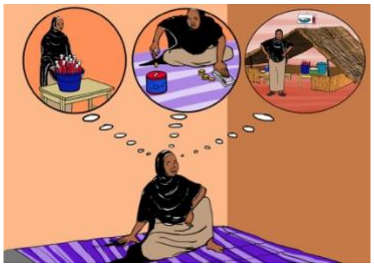	Amina developed her activities by being innovative, meaning she thought of new things other people had not thought of. She was creative, meaning she changed her activities.
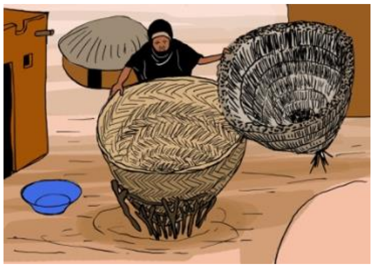	She was persistent in developing her activities and overcoming hardships, such as low yields after a drought. She sought out new ideas and strategies when she felt stuck.

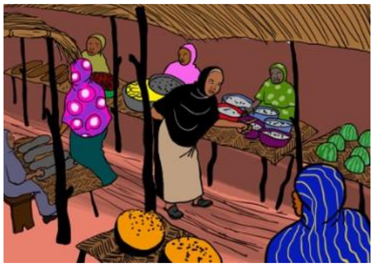	She was also strategic. She looked around the market to see which activity would be profitable based on what customers wanted in the market and on how her business could be unique. She was determined to sell bissap juice.
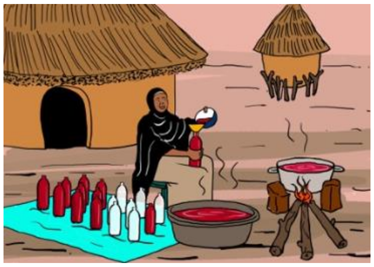	She was proactive and took the initiative to seek out a way to learn how to make bissap juice.
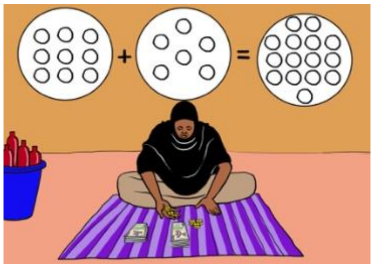	She planned for the future by saving. She made calculations on how her savings could be used to develop her activities. With her savings, she was able to re-invest in her activities and start new ones.
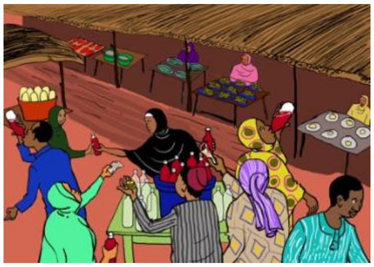	She worked hard in her activities to increase her profits. Through her self-initiative and passion, she became a successful business woman.
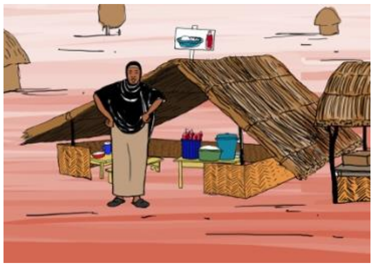	In the end, Amina opened her own store. She was able to achieve her economic goals and to become a model female entrepreneur. She also was able to advance her financial security and her socioeconomic status.

Guided exercise:

- Do you think that Amina was a successful female entrepreneur? What steps did she take to develop her activities? (Prompt: For example, what did she do with her money?) (Describe 2-5 actions)
- Which actions of a model female entrepreneur did she demonstrate? (Describe 2-5 actions)
- Amina was able to become a successful entrepreneur by saving and developing her activities. Now think about yourself and your future. Imagine you were to successfully develop your

activities, like Amina did, over the course of the safety net program. In one year, what do you envision you will have in terms of resources or do differently in terms of your activities? Which changes in your financial situation and activities would you like to carry out that you think are also possible? (Prompt: For example, what products could you sell? How much would you have in savings?)
    - Goal 1:
    - Goal 2:
- How would you feel if these goals were achieved? (Describe 1-2 feelings)
- Entrepreneurs often face obstacles while developing their activities, such as lack of start-up funds or market access difficulties. For example, Amina had low yields in millet production one year. These obstacles can be difficult and could hurt your morale. What could make it difficult to grow your business?
    - Obstacle 1:
    - Obstacle 2:
- How would these obstacles impede the realization of your goals for your activities? Can you describe them in greater detail?
- What alternative strategies could you take if you encounter these two obstacles? Let's start with the first obstacle you identified: (INSERT OBSTACLE 1 HERE). What is one strategy you could use to overcome this obstacle? We want to fill in the sentence: "If I meet this obstacle of ___, I will ___." (Prompt: For example, what new products could you buy, how can you be persistent, what new information might you be looking for that others do not know?)
    - Thus, to summarize, "If I meet this obstacle of (INSERT OBSTACLE 1 HERE), I will (INSERT STRATEGY TO OVERCOME OBSTACLE 1 HERE)."
- And now, the second obstacle you have described: (INSERT OBSTACLE 2 HERE). What is a strategy you could use to overcome this second obstacle?
    - Thus, to summarize, "If I meet this obstacle of (INSERT OBSTACLE 2 HERE), I will (INSERT STRATEGY TO OVERCOME OBSTACLE 2 HERE)."

Relational Agency.

Introduction: The business and life skills training is intended to help you make changes so you can better help your family and your village. The goal of the program is to help families grow together and to support each other. Through the business skills training and other measures, the program aims to help women across Niger advance their families' wellbeing, better cope with shocks in the future, and to have greater peace in their households and their communities.

Film script by scene:

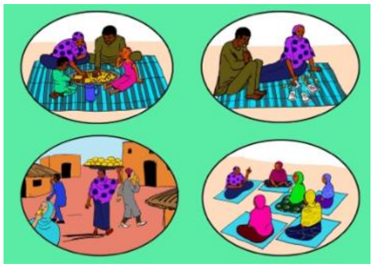	In the movie you saw, the woman envisioned a plan for helping advance her family's health and wellbeing. She developed her activities and started saving. By doing this, she was able to keep her children in school and grow her household's activities. She also helped other women in her village by teaching them new skills and sharing new information.

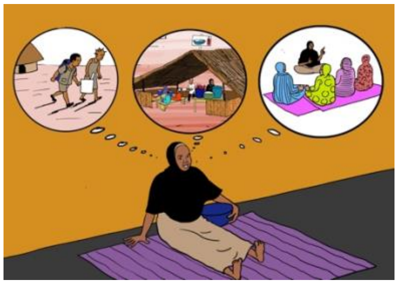	
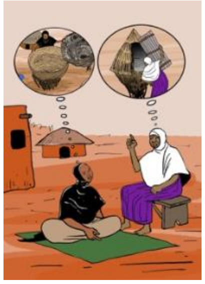	Amina developed her activities by seeking advice from others, such as from her mother. She learned that her mother had adapted her activities in the past during crises, and she learned she should do the same.
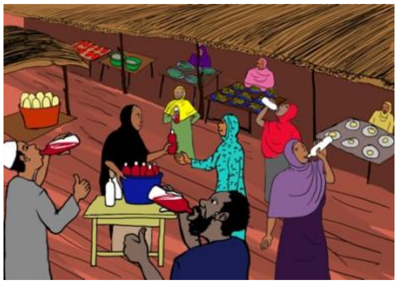	To determine which new activity to start, she observed which products people in her village would enjoy -- bissap juice after their meals.
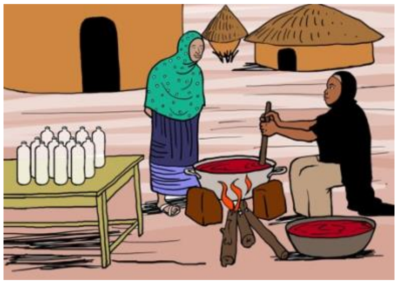	Then, she reached out to her cousin to learn to make bissap juice. Later, she passed along this knowledge to others.
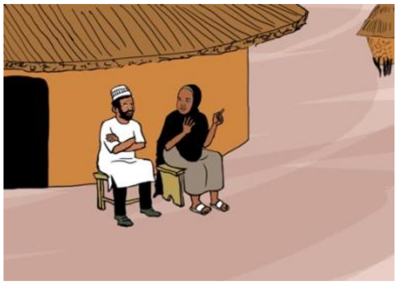	At first her husband was resistant to her saving with her women's group. However, she talked with him about how saving could help them keep their children in school and grow his business as well.

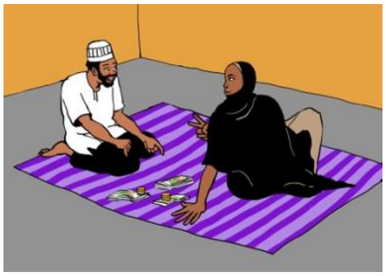	Her father-in-law also helped mediate the conflict and advised the couple to support each other. It was through this initial conflict that Amina and her husband were able to achieve greater peace in the long term.
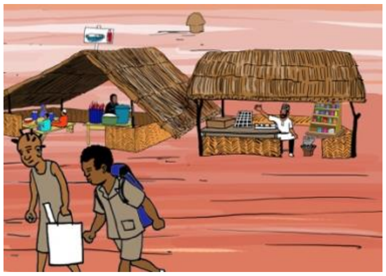	In the end, using her earnings and savings, she and her husband were able to develop their activities and to open a store together. They also were able to send their children to school.
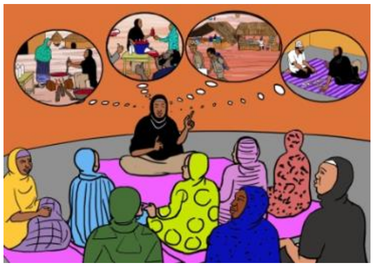	As she had been taught, Amina passed along new knowledge and skills she learned to other women in her village. In this way, she modeled generosity and respect to her children and other women in her village.

Guided exercise:

- Do you think that Amina respected her family and her village? (Pause) If so, how? (Describe 2-5 ways)
- Did Amina help her family and her village? (Pause) If so, how? (Describe 2-5 ways)
- Amina was able to better support her family and her village through saving and developing a new activity. Now think about yourself and your family's future. Imagine you and your family were to successfully develop your activities like Amina did, over the course of the safety net program. In one year, what do you envision you will have in terms of resources or do differently in terms of your activities? Which changes in your financial situation and activities would you like to carry out that you think are also possible? (Prompt: For example, what products could you sell? How much would you have in savings?)
    - Goal 1:
    - Goal 2:
- How will this help others you care about in your family, in your village? Who will you help and how? (Prompt: for who (e.g. your children, your parents, your women's group), for how (e.g. school fees, food, teaching new skills))
    - Person/group 1:
        - How?
    - Is there someone else this would help?
Person/group 2:
        - How?
- Amina's husband was initially resistant to her ideas for savings and growing their activities. However, she talked with him about how it could help them keep their children in school and grow her husband's business. Like Amina, women across Niger are increasingly working in

collaboration with their husbands and their families to advance their activities and to achieve greater peace in their households in the long term.
If a woman's husband or other family member is resistant to accepting a woman's proposals for her activities (for example, starting new activities), what advice would you give this woman to better communicate with them? For example, what could she say to this person? (Describe 1-2 tips of advice)

- When conflicts arise, women often seek advice and encouragement from those close to them. If you had a conflict in your household related to your economic activities, who could you talk to for such advice and encouragement? For example, this might be a coach, a parent, or a friend. Who is this person in relation to you?
- To grow their business, Amina, her family (her mother, cousin) and her savings group learned from each other, and encouraged each other. What are the ways in which the women in your family and community are able to help each other? (Describe 1-3 ways)
- Women across Niger will be participating in this program and will have the opportunity to become models for their children and their communities. Which values or behaviors do you want you and your group to model for your children and to younger women in your village? (Prompt: For example, solidarity, generosity, respect)

Measures.

Post-intervention Survey Measures.

Responses to self-reported psychosocial measures and hypothetical economic scenarios were collected among those in intervention conditions only.

Note that many of these measures were original, meaning that they were developed to be specific to this economic and cultural context. Given time constraints, we were unable to assess the properties of these measures before the survey, and several were found post-hoc to have poor properties (e.g., low internal reliability, low variation, ceiling effects). While we pre-registered a split sample analytic approach due to the exploratory nature of these measures and to help control the rate of false discovery (11), we ultimately found no significant effects on any outcome measure and thus present analyses on the full sample. To create composites, variables were averaged together.

Economic outcomes. Three composite variables (*approach behaviors*, *approach feelings*, and *budget allocation*) comprise an economic composite index, which relates to business intentions and investment behaviors. *Approach behaviors* and *approach feelings* assess response to 4 hypothetical business decisions that relate to: reinvesting in a profitable activity following an intrahousehold disagreement, becoming a traveling saleswoman, seeking information on business development, and asking trainers for advice on their business. Different options were given for each scenario but approach behaviors were classified into binary indicators (approach/avoid) and then summed. For each of the 4 scenarios, respondents were asked how they would feel on a 4-point scale for 4 *feelings* ($\alpha = 0.56$): confident vs. uncertain, proud vs. ashamed, generous vs. selfish, and harmonious vs. conflictual. A hypothetical *budget allocation* asked respondents about a scenario in which they had 6,500 CFA in surplus business profits and asked them how much they would invest in savings and in her business (as compared to food, school fees, community projects or other self-described purchases). *Optimistic program expectations* asked respondents 4 prediction questions ($\alpha = 0.70$) on how many out of 10 program participants would increase their profits under different scenarios and how many would attend all life skills and business training sessions.

Relational outcomes. A relational composite index was comprised of 5 composite variables (*social standing, social norms, social support, anticipation of negative reputation, and trust*) ($\alpha = 0.36$). *Social standing* uses a 10-point ladder for 5 questions ($\alpha = 0.84$) about women's current subjective social status, how well-regarded they are in their community, how much their opinion is followed and whether that will increase or decrease in the future or stay the same in the future, and how much they feel they are a person who models good moral character. *Social norms* ($\alpha = -0.06$) relate to women's economic activities and ask them to estimate out of 10 women how many would be supported by their parents to become traveling saleswomen, how many of 10 would advocate for their control over their earnings in the household, and how many of 10 would be given loans if requested. *Social support* ($\alpha = 0.12$) asks women on a 4-point scale how much they feel they can count on financial help from women in their savings group

and to estimate out of 10 women how many would give them money if requested. *Trust* is measured with a single item "Out of 10 people in your region, how many people are good and trustworthy vs. bad and untrustworthy?". For *anticipation of negative reputation,* participants were asked how they think they will be seen by others in their family and community as they develop their businesses and were given open-ended response options, two for family and two for community; responses were then classified by enumerators as positive, negative, or unclear, and the final variable was a binary indicator of whether they reported anticipating any negative consequences across these questions.

Personal outcomes. A personal composite index was comprised of two variables (α = 0.20). *Future expectations (SES)* included 2 items (α = 0.46) asking about their anticipated socioeconomic status in two years and that of their youngest child or grandchild when they become 30 (10-point ladder). *Self-efficacy* assessed 3 items (α = 0.63): respondent's perceived ability to adapt to difficulties, cope with unexpected events and do things as well as most people, each on 4-point scales.

Prosocial preferences gave respondents two hypothetical allocation tasks. The first asked them to allocate 170,000 CFA of an NGO to community projects versus individual households. The second asked them how much of 6,500 CFA in profits they would give to fund community projects.

Amina evaluation assessed respondents' evaluations of the role model displayed in the intervention materials on a 10-point scale, specifically ratings of her morality, respect, social standing, and economic mobility (α = 0.78).

Endline Survey Measures.

The endline survey assessed economic outcomes and psychosocial outcomes relating to personal and relational factors. Due to budget and logistical constraints, this 45-minute endline survey was an abbreviated and slightly altered version of the measures used in Study 2. The construction of these outcomes aligns to the extent possible with Study 2 and as reported in Bossuroy et al. (2022). We construct composite indices across all outcomes within each type (economic, personal, and relational). All outcomes and outcome indices are standardized to the control group's mean and standard deviation (12).

Economic outcomes. The *food security index* is a composite of two constructs. *Household food security frequency* over the past 12 months is assessed with two items adapted from the Food Insecurity Experience Scale (FIES, e.g., having gone a whole day without food, 0=Almost every month to 4=No, never) (13). *Dietary diversity* is captured with an abbreviated measure of food consumption; it is a weighted sum of the number of days in the past week that the respondent has eaten vegetables and meat, where meat is given a weight of four and vegetables of one (14).

Business engagement index assesses the number of off-farm businesses owned or operated in the past 12 months by the program participant, the number started in the last 12 months, the number of businesses the beneficiary intends to expand (vs maintain or abandon), and the total value of business assets and business investments. This index also included three additional variables than those pre-registered in order to match the construction of the business outcomes index in Bossuroy et al. (2022): the sum of days worked on all businesses in the past month, having an off-farm business (or not), and an index of self-reported healthy business practices (e.g., keeping a sales ledger). *Business performance index* assesses the total value of profits and revenues across all beneficiary owned businesses.

Psychosocial outcomes. We construct a summary *personal composite index* across three self-oriented constructs: subjective well-being, self-efficacy, and future expectations (α = 0.48). We measure *subjective well-being* with 14 standardized items (α = 0.74): ten items from the CESD-R-10 depression screener (0-7 days, e.g., "Over the past seven days, how often have you felt depressed?", *reverse-coded*), a life satisfaction item (Cantril ladder, 10-point scale), a novel measure of inner peace (10-point scale), an original measure of feeling blessed by God (1=No, definitely not to 4=Yes, definitely), and *subjective health* (1=Poor to 5=Excellent). *Self-efficacy* (α = 0.70) is assessed with 4 items from the Generalized Self-Efficacy Scale (15) and one item from the Rosenberg Self-Esteem Scale (1=Not at all to 4=Yes, absolutely) (16). *Future expectations* is a single item of expected socioeconomic standing in two years, using the MacArthur Scale of Subjective Social Status (10-point scale) (17).

Relational outcomes. We construct a summary *relational composite index* across six other-oriented constructs (α = 0.49). We measure *social standing* with 3 items (α = 0.68): the MacArthur Scale of Subjective Social Status plus two adapted such scales assessing how much respondents feel that they are respected in society and are a person of good moral character (10-point scale). *Social and financial support* is assessed with 4 items (α = 0.63) asking how many people the respondent could ask for advice on their economic activity inside their household and in the community, how many people they could ask

for advice if they experience an interpersonal conflict, and their perceived probability of being able to amass a small sum of money in emergencies (1=Not at all likely to 4=Very likely). *Social cohesion and community closeness* is assessed with 4 items (α = 0.14): how many people out of ten in the village the respondent feels they can trust (10-point scale), how many enemies (someone who "wishes you to fail or would try to sabotage their progress") they perceive they have (1=No one to 4=A lot), how much they feel it is their duty to sacrifice for their community even at their own expense (1=No, not at all to 4=Yes, definitely), and how close they feel toward their community (4-point scale of Inclusion of Other in Self (IOS) Scale) (6).

We assess *control over earnings* with 4 items (α =0.61): three capturing how much women feel that their opinion matters in decisions related to their own earnings, daily spending, and non-agricultural businesses (1=Does not matter at all to 3=Matters a lot) and whether the household has prevented the respondent from working outside the home in the last 12 months (0=Yes, 1=No). *Partner dynamics* was captured with 2 items (α = 0.68): one assessing how close respondents feel towards their partner (4-point, IOS scale) and one assessing how comfortable they feel telling their partner that they disagree with them (1=Never to 4=Most of the time). *Household interpersonal dynamics* was captured with 3 items (α = 0.38) assessing how close respondents feel towards their household (4-point, IOS scale), how respected they feel by their household in regards to their economic activity (1=No, not at all to 4=Yes, a lot), and how much they have experienced household tension in the last 6 months (1=No, not at all to 4=Yes, a lot).

Other. We assess *redistributive preferences* with 3 exploratory items assessing sharing-oriented attitudes and behaviors (how much alms respondents have given to the less fortunate in their community in the last two months, what percentage of any extra yields would they share with others in their community as opposed to save for themselves, and preferences for the village to develop together versus separately), yet these did not hang together well (α = 0.00).

Sociodemographics. Most sociodemographic measures including Proxy Means Targeting (PMT) poverty score (Premand & Schnitzer, 2021), age, relationship to household head, and nomad status were collected in a census survey prior to the randomization.

Section S2. Additional Analyses

We did pre-register analyses on administrative data for participation rates in the business and life skills trainings that occurred shortly after the delivery of these agency interventions and found no difference across conditions. This is very likely due to ceiling effects, given that the median number of sessions attended by women in all conditions was 12 of 12 sessions offered ($M_{Control}$=10.81; personal: β=-0.09, t(2622)=-0.71, p=0.480; relational: β=-0.07, t(2622)=-0.62, p=0.538).

In addition to the randomization to the control or one of two treatment arms, we also randomized the proportion of individuals within a village (25%, 50%, or 75% saturation) to be treated. Table S9 and Table S11 display results from that village-level randomization on psychosocial and economic outcomes. We predominantly find no significant differences across saturation levels, with only a few marginally positive results on select psychosocial outcomes.

In terms of the mechanisms of Study 3, one question pertains to whether the interventions caused changes in psychosocial processes and/or in the objective ways that participants conducted business. On the former, we do find evidence for impacts of the *relational agency* intervention on psychosocial processes related to personal motivation and relational processes, as presented in Study 3. On the latter, we do not find evidence of differences in how women ran their businesses. For instance, those in the either agency condition were not more collaborative in their businesses according to the number of collaborators they had (control: M=0.24 collaborators) (relational: β=-0.12, t(2474)=-0.29, *p*=.772; personal: β=0.04, t(2474)=0.95, *p*=.342).

Section S3. Pre-Registration Deviations for Study 3

Endline Survey.

1. Internal validity
   a. We were ultimately unable to obtain the variable of "household size" for the full sample and thus do not check it for balance across conditions. However, we were able to add a variable

indicating whether a household lives in a hamlet on the outskirts of a village or inside the village as an additional sociodemographic variable. We use this variable for randomization balance checks (as was pre-registered in the immediate outcomes survey) and for differential attrition checks.

   b. There is an imbalance in the share of participants sampled for the main policy experiment, which is higher for the two treatment conditions compared to the control ($M_{Control}$=13%; personal: M=23%. Relational: M=22%, p<.001). This was due to a coding error. Notably, however, these participants had been randomly sampled from villages and thus should not differ in meaningful ways from the non-sampled. In addition, a binary variable indicating inclusion in the policy experiment sample had been pre-registered as a covariate and thus is included in all analyses, which also helps address the observed imbalance.

2. Empirical strategy
   a. Because we found differing patterns of results for each of the psychosocial intervention conditions, compared to the control, and because our primary hypotheses specified different patterns of results for each condition compared to the control, we present comparisons of each psychosocial condition to the control and do not present analyses collapsing across the two psychosocial conditions.
   b. Cluster robust SEs were pre-registered because due to assumptions of non-independence given the group-based structure of the ASP program. However, our experiment's randomization was conducted at the individual level and not the group level. Given these design considerations and recent guidance on when not to cluster standard errors (18), we ultimately prioritize robust SEs instead of cluster robust SEs for interpretation purposes. We present both types of p values and consider the cluster robust p-values to be conservative estimates.
3. Outcomes construction
   a. The most meaningful changes to outcome measure constructions were made in order to match, to the extent possible, the final constructions of variables used in the analyses presented in Bossuroy et al. (2022) (19). Beyond that:
      i. We did not analyze savings due to ambiguity in the wording of the questions, specifically which types of savings groups they referred to in the context of multiple ongoing programs in the villages. In addition, accurate savings could not be calculated due to the complicated structure of different types of savings groups and the omission of relevant questions on those structures (e.g., frequency of meeting, savings per meeting).
      ii. We prioritized administrative data on program participation and thus do not include analyses of self-reported participation.
      iii. For dietary diversity, we did not ultimately include a measure of fruit consumption in the endline survey and thus it is not in the analysis.
      iv. For the exploratory Type B "prosociality" measure, we prioritized two allocation tasks items that could be clearly interpreted and that did not suffer from low variation or ceiling effects.
4. Given that rate of missingness was low, we did not use imputation, and we use all available data to construct indices (i.e., using one variable even if others are missing for an index).
5. We found limited evidence of heterogeneity on primary pre-registered variables nor on other exploratory variables. Results of analyses on the primary and many exploratory variables are available in the open-source code and data and may be examined by other researchers.

Immediate Outcomes. This pertains to the survey conducted only with those in psychosocial treatment conditions immediately after intervention delivery. While we pre-registered a split sample analytic approach due to the exploratory nature of these measures and to help control the rate of false discovery (11), we ultimately found no significant effects between the *personal agency* and *relational agency* intervention conditions on any outcome measure, except a marginal effect on one of our three confirmatory hypotheses related to negative reputational consequences. Thus, we present analyses on the full sample (see Table S9 and Additional Analyses). To be consistent with other analyses, we do not include saturation of treatment assignment as a covariate, and we apply two-sided tests rather than one-sided tests to be conservative.

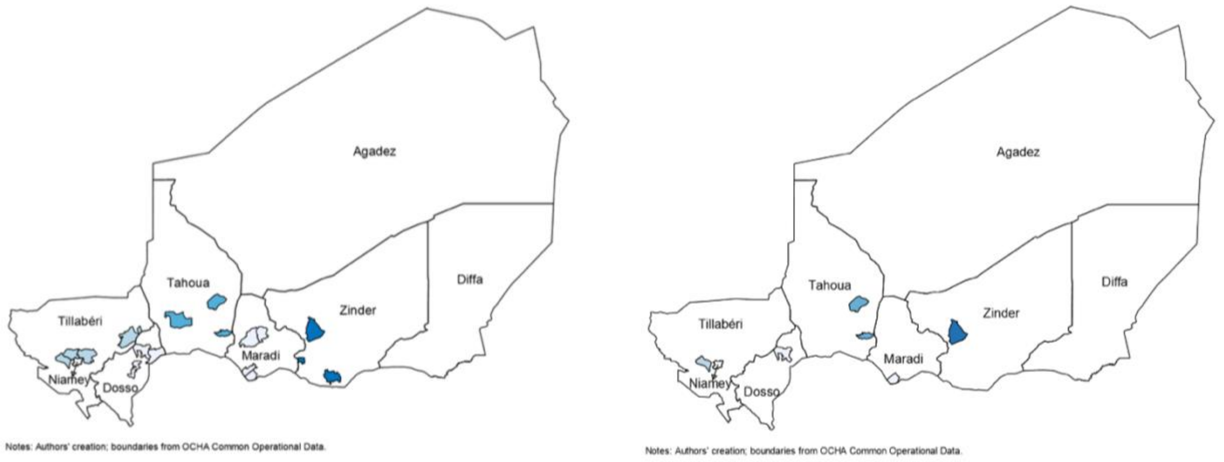

Figure S1. Map of communes in the policy experiment (left) and Study 3 (right) samples

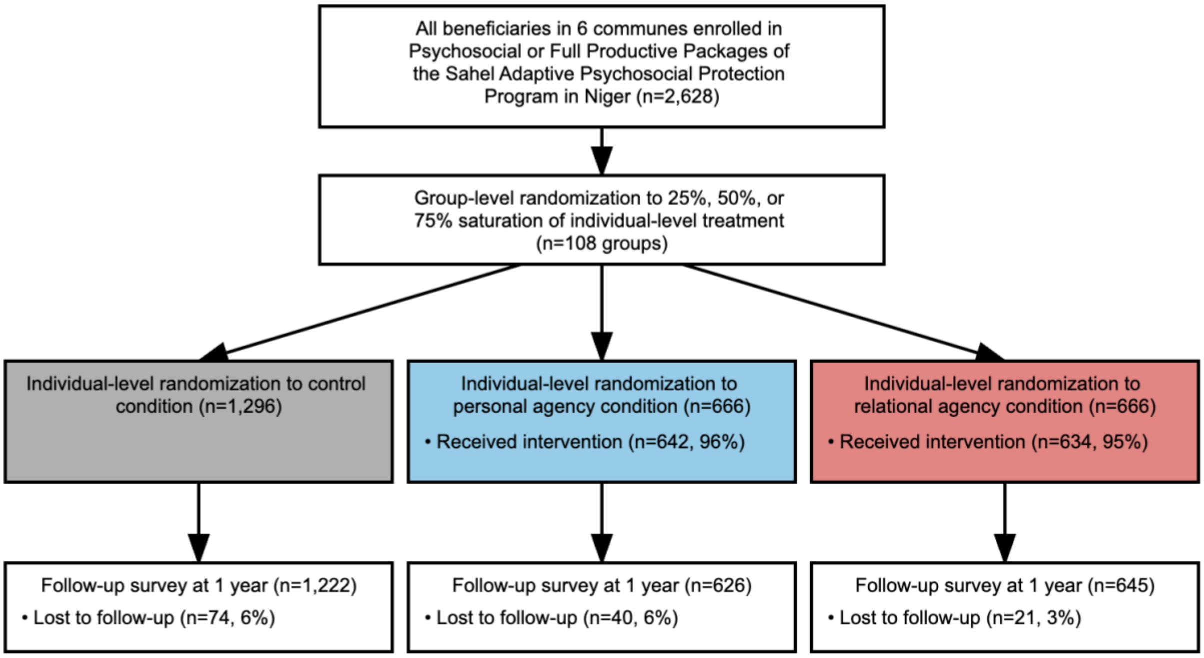

Figure S2. Flowchart of the experimental design of Study 3
Within the policy experiment, the Full and Psychosocial arms included psychosocial interventions (specifically community sensitization and life skills training), regular cash transfers, savings groups formation, group coaching, and micro-entrepreneurship training. The Full arm added a lump-sum cash grant. Randomization strata included policy experiment treatment arm, timing of the program components by season, and participation in the policy experiment baseline survey.

Table S1. Exploratory factor analysis of psychosocial variables in Study 2

Theoretical classification	Variable	Factor 1	Factor 2
Personal	Self-Efficacy	**.40**	**.32**
Personal	Future Expectations	**.72**	
Personal	Mental Health	**.54**	
Relational	Collective Action	.16	**.21**
Relational	Social Standing	**.73**	
Relational	Social Support	.15	.15
Relational	Social Norms		**.27**
Relational	Intra-Household Dynamics		**.21**
Relational	Financial Support	.10	**.21**
Relational	Social Cohesion	.11	**.24**
Relational	Controls Earnings		**.66**
Relational	Control in Household		**.67**

Note. Data is from the 6-month follow-up of the policy experiment, excluding observations with missings (N=4,242). Loadings ≥ .20 are in bold. Extraction used oblimin rotation. Factor 1 eigenvalue = 1.58 (13% variance), Factor 2 eigenvalue = 1.28 (11% variance). Cumulative variance explained = 24%. Classified personal variables load primarily on the first factor and classified relational variables loaded primarily on the second, with a few variables showing cross-loadings or deviations from this pattern (e.g., social standing which shared a similar response scale with the future expectations measure).

Table S2. Correlation matrix among psychosocial variables in the policy experiment's Psychosocial arm at the 6-month follow-up (Study 2)

	Social_Standing	Controls_Earnings	Financial_Support	Social_Support	Social_Norms	Social_Cohesion	Intra.Household_Dynamics	Control_in_HH	Self.Efficacy	Future_Expectations	Mental_Health
Future_Expectations											0.34
Self.Efficacy										0.27	0.24
Control_in_HH									0.19	0.07	0.01
Intra.Household_Dynamics								0.12	0.08	0.09	0.07
Social_Cohesion							0.24	0.13	0.2	0.02	0.14
Social_Norms						0.06	0.03	0.21	0.12	0.02	0.06
Social_Support					0.05	0.09	0.02	0.08	0.11	0.12	-0.01
Financial_Support				0.2	-0.01	0.25	0.08	0.01	0.19	0.03	0.1
Controls_Earnings			0.05	0.13	0.15	0.04	0.15	0.58	0.16	0.06	0
Social_Standing		0.07	-0.01	0.14	0.06	0.05	0.04	0.06	0.26	0.55	0.35
Collective_Action	0.09	0.03	0.15	0.15	0.03	0.18	0.03	0.09	0.2	0.13	0.03

Table S3. Balance check of key variables across conditions in Study 3

	**Control (N=1296)**	**Personal agency (N=666)**	**Relational agency (N=666)**	**Total (N=2628)**	**p value**
Proxy Means Testing Score					0.487
Mean (SD)	12.26 (0.31)	12.25 (0.33)	12.25 (0.31)	12.26 (0.31)	
Age					0.964
Mean (SD)	34.33 (14.10)	34.39 (13.78)	34.51 (14.01)	34.39 (13.99)	
Is head of household					0.660
Mean (SD)	0.12 (0.32)	0.13 (0.34)	0.12 (0.33)	0.12 (0.33)	
Is nomad					0.733
Mean (SD)	0.10 (0.30)	0.11 (0.32)	0.11 (0.31)	0.11 (0.31)	
Lives in a hamlet					0.976
Mean (SD)	0.22 (0.42)	0.22 (0.42)	0.22 (0.41)		
Policy experiment treatment arm					0.955
Full N (%)	782 (60.3%)	398 (59.8%)	398 (59.8%)	1578 (60.0%)	
Psychosocial N (%)	514 (39.7%)	268 (40.2%)	268 (40.2%)	1050 (40.0%)	
Timing of programs in policy experiment					0.549
Early N (%)	729 (56.2%)	361 (54.2%)	360 (54.1%)	1450 (55.2%)	
Late N (%)	567 (43.8%)	305 (45.8%)	306 (45.9%)	1178 (44.8%)	

The p values reflect an ANOVA or chi-squared test (depending on the variable type) comparing each variable across treatment and control conditions.[1]

[1] Note that there is an imbalance in the share of participants sampled for the main policy experiment, which is higher for the two treatment conditions ($M_{Control}$=13%; personal: M=23%; relational: M=22%, p<.001). This was due to a coding error. Notably, all participants had been randomly sampled from villages and thus should not differ in meaningful ways from the non-sampled individuals. All analyses control for a binary variable indicating inclusion in the policy experiment.

Table S4. Check for association of attrition with baseline sociodemographics in Study 3

**Outcome**	**df**	**Beta**	**SE**	**P value**
Personal agency Condition	2623	0.16	0.76	0.446
Relational agency Condition	2623	-0.52	0.25	0.038*
PMT	2623	0.55	0.35	0.121
Age	2623	0.01	0.01	0.025*
Is head of household	2623	0.50	0.23	0.030*
Is nomad	2623	0.05	0.29	0.865
Lives in a hamlet	2623	-0.27	0.23	0.243

Coefficients are from logistic regressions regressing attrition on each variable. Regressions control for randomization strata and standard errors are robust. †p<.10, *p<.05, **p<.01, ***p<.001.

Table S5. Impacts of personal agency and relational agency interventions on economic outcomes at endline in Study 3

		**Control**	**Personal agency**	**Relational agency**
**Outcome**	df	Mean<br>(SD)	**Coefficient**<br>(SE)<br>Robust *p*-value<br>Cluster robust *p*-value	**Coefficient**<br>(SE)<br>Robust *p*-value<br>Cluster robust *p*-value
**Economic Composite Index**	**2473**	**0.00**<br>**(1.00)**	**0.07**<br>**(0.05)**<br>**0.169**<br>**0.264**	**0.12**<br>**(0.05)**<br>**0.013***<br>**0.023***
**Food Security Index**	**2473**	**0.00**<br>**(1.00)**	**0.07**<br>**(0.05)**<br>**0.152**<br>**0.262**	**0.11**<br>**(0.05)**<br>**0.029***<br>**0.059†**
Food security	2473	6.83<br>(1.52)	0.15<br>(0.07)<br>0.034*<br>0.074†	0.16<br>(0.07)<br>0.032*<br>0.054†
Dietary diversity	2473	9.12<br>(8.75)	0.07<br>(0.42)<br>0.870<br>0.898	0.59<br>(0.43)<br>0.169<br>0.214
**Business Omnibus Index**	**2474**	**0.00**<br>**(1.00)**	**0.04**<br>**(0.05)**<br>**0.386**<br>**0.466**	**0.09**<br>**(0.05)**<br>**0.071†**<br>**0.108**
**Business Engagement Index**	**2474**	**0.00**<br>**(1.00)**	**0.05**<br>**(0.05)**<br>**0.290**<br>**0.342**	**0.09**<br>**(0.05)**<br>**0.055†**<br>**0.095†**
Has a business	2474	0.82<br>(0.38)	-0.01<br>(0.02)<br>0.778<br>0.779	0.03<br>(0.02)<br>0.079†<br>0.118
No. businesses	2474	1.24<br>(1.21)	0.01<br>(0.06)<br>0.848<br>0.856	0.05<br>(0.06)<br>0.370<br>0.410
No. businesses past year	2474	0.48<br>(1.04)	0.04<br>(0.05)<br>0.395<br>0.393	0.05<br>(0.05)<br>0.295<br>0.331
Business investments (yearly, USD)	2474	106.12<br>(181.94)	19.78<br>(9.17)<br>0.031*<br>0.059†	14.22<br>(8.85)<br>0.108<br>0.163
Business assets value (yearly, USD)	2474	15.02<br>(22.19)	0.62<br>(1.13)<br>0.585<br>0.629	0.90<br>(1.09)<br>0.409<br>0.474

No. days worked	2474	17.47<br>(20.15)	0.78<br>(0.94)<br>0.407<br>0.497	0.82<br>(0.90)<br>0.366<br>0.402
Growth intentions	2474	1.06<br>(0.83)	0.01<br>(0.04)<br>0.750<br>0.761	0.07<br>(0.04)<br>0.087†<br>0.119
Healthy business practices index	2457	0.00<br>(1.00)	0.06<br>(0.05)<br>0.259<br>0.281	0.06<br>(0.05)<br>0.215<br>0.253
**Business Performance Index**	**2474**	**0.00<br>(1.00)**	**0.00<br>(0.05)<br>0.943<br>0.955**	**0.05<br>(0.05)<br>0.354<br>0.377**
Business profits (monthly, USD)	2474	35.61<br>(50.24)	-0.49<br>(2.44)<br>0.841<br>0.876	2.91<br>(2.53)<br>0.249<br>0.297
Business revenues (monthly, USD)	2474	118.45<br>(162.20)	2.68<br>(7.88)<br>0.734<br>0.783	5.01<br>(7.86)<br>0.524<br>0.518

Note: Columns 4 and 5 show regression output comparing the *personal agency* and *relational agency* conditions, respectively, to the control condition in Study 3. Regressions control for randomization strata and standard errors are robust. Data on beneficiary businesses was collected on a subset of common off-farm businesses types. †p<.10, *p<.05, **p<.01, ***p<.001.

Table S6. Robustness analyses: Impacts of personal agency and relational agency interventions on economic and psychosocial outcome indices, controlling for age and head of household status, in Study 3

		**Control**	**Personal agency**	**Relational agency**
**Outcome**	df	Mean<br>(SD)	**Coefficient**<br>(SE)<br>Robust *p*-value<br>Cluster robust *p*-value	**Coefficient**<br>(SE)<br>Robust *p*-value<br>Cluster robust *p*-value
Economic Composite Index	2472	0.00<br>(1.00)	0.07<br>(0.05)<br>0.168<br>0.263	0.12<br>(0.05)<br>0.013*<br>0.022*
Food Security Index	2471	0.00<br>(1.00)	0.07<br>(0.05)<br>0.150<br>0.260	0.11<br>(0.05)<br>0.029*<br>0.059†
Business Omnibus Index	2472	0.00<br>(1.00)	0.04<br>(0.05)<br>0.387<br>0.467	0.09<br>(0.05)<br>0.071†<br>0.107
Psychosocial composite index	2485	0.00<br>(1.00)	0.10<br>(0.05)<br>0.039*<br>0.058†	0.12<br>(0.05)<br>0.014*<br>0.029*
Personal composite index	2485	0.00<br>(1.00)	0.14<br>(0.05)<br>0.003**<br>0.010*	0.12<br>(0.04)<br>0.009**<br>0.023*
Relational composite index	2485	0.00<br>(1.00)	0.05<br>(0.05)<br>0.333<br>0.337	0.09<br>(0.05)<br>0.072†<br>0.118

Note: Columns 4 and 5 show regression output comparing the *personal agency* and *relational agency* conditions, respectively, to the control condition in Study 3. Regressions control for randomization strata, age, and head of household status. Standard errors are robust. †p<.10, *p<.05, **p<.01, ***p<.001.

Table S7. Impacts of personal agency and relational agency interventions on household-owned and -managed businesses among a subsample of participants in the policy experiment and Study 3

		Control	Personal agency	Relational agency
**Outcome**	df	Mean<br>(SD)	**Coefficient**<br>(SE)<br>Robust *p*-value<br>Cluster robust *p*-value	**Coefficient**<br>(SE)<br>Robust *p*-value<br>Cluster robust *p*-value
**Business Omnibus Index (HH)**	**452**	**0.00**<br>**(1.00)**	**0.19**<br>**(0.13)**<br>**0.142**<br>**0.128**	**0.30**<br>**(0.13)**<br>**0.023***<br>**0.009****
**Business Engagement Index (HH)**	**452**	**0.00**<br>**(1.00)**	**0.19**<br>**(0.13)**<br>**0.151**<br>**0.154**	**0.28**<br>**(0.14)**<br>**0.039***<br>**0.021***
HH has a business	452	0.51<br>(0.50)	-0.02<br>(0.06)<br>0.769<br>0.757	0.06<br>(0.06)<br>0.286<br>0.236
No. HH businesses	452	0.64<br>(0.75)	0.07<br>(0.1)<br>0.435<br>0.403	0.18<br>(0.1)<br>0.069†<br>0.053†
No. HH businesses past year	452	0.14<br>(0.42)	0.05<br>(0.05)<br>0.312<br>0.334	0.02<br>(0.05)<br>0.661<br>0.668
HH Business investments (yearly, USD)	452	0.50<br>(6.40)	1.71<br>(1.11)<br>0.125<br>0.131	1.65<br>(1.16)<br>0.156<br>0.157
HH Business asset value (USD)	452	50.90<br>(180.96)	-10.64<br>(18.34)<br>0.562<br>0.574	15.95<br>(24.56)<br>0.516<br>0.539
No. days worked (HH)	452	5.62<br>(10.88)	1.1<br>(1.32)<br>0.406<br>0.434	1.74<br>(1.3)<br>0.182<br>0.193
Growth intentions (HH)	452	0.01<br>(0.08)	0.03<br>(0.02)<br>0.084†<br>0.086†	0.03<br>(0.02)<br>0.083†<br>0.074†
**Business Performance Index (HH)**	**452**	**0.00**<br>**(1.00)**	**0.11**<br>**(0.13)**<br>**0.400**<br>**0.381**	**0.21**<br>**(0.13)**<br>**0.099†**<br>**0.056†**
HH Business profits (monthly, USD)	452	31.71<br>(80.83)	10.68<br>(10.77)	14.81<br>(10.18)

			0.322<br>0.301	0.146<br>0.103
HH Business revenues (monthly, USD)	452	69.71<br>(184.59)	13.66<br>(21.56)<br>0.527<br>0.512	41.25<br>(24.53)<br>0.093†<br>0.053†

Note: Columns 4 and 5 show regression output comparing the *personal agency* and *relational agency* conditions, respectively, to the control condition in Study 3. Regressions control for randomization strata and standard errors are robust. This data is from the subsample randomly selected to participate in the policy experiment, in which more data was collected from beneficiaries' households. Data was only collected on a subset of common off-farm businesses types. "HH" stands for household. †p<.10, *p<.05, **p<.01, ***p<.001.

Table S8. Effects of relational agency intervention versus personal agency intervention immediately post-intervention in Study 3

		Personal agency	Relational agency
**Outcome**	df	**Mean**<br>(SD)	**Coefficient**<br>(SE)<br>Robust *p*-value
**Economic Composite Index**	**1271**	**0.96**<br>**(0.15)**	**0.00**<br>**(0.01)**<br>**0.657**
Approach behaviors (hypothetical scenarios)	1271	2.72<br>(0.94)	0.06<br>(0.05)<br>0.277
Approach feelings (hypothetical scenarios)	1271	3.70<br>(0.33)	-0.01<br>(0.02)<br>0.656
Budget Allocation (hypothetical scenario)	1271	7.17<br>(2.04)	-0.04<br>(0.12)<br>0.704
**Relational Composite Index**	1327	**0.95**<br>**(0.15)**	**0.01**<br>**(0.01)**<br>**0.176**
Social Standing	1271	7.07<br>(1.59)	0.00<br>(0.09)<br>0.985
Social Norms	1271	6.49<br>(1.37)	-0.02<br>(0.08)<br>0.797
Social Support	1271	0.94<br>(0.24)	0.01<br>(0.01)<br>0.252
Anticipation of Negative Reputation (hypothetical scenarios)	1327	0.15<br>(0.36)	-0.03<br>(0.02)<br>0.088†
Trust	1271	6.54<br>(2.49)	0.03<br>(0.14)<br>0.813
**Personal Composite Index**	1327	8.65<br>(2.04)	-0.08<br>(0.12)<br>0.498
Self-efficacy	1327	8.97<br>(2.53)	-0.09<br>(0.14)<br>0.541
Future expectations (SES)	1271	8.64<br>(1.34)	0.03<br>(0.08)<br>0.677
**Other**			
Future Expectations (Program)	1271	7.29<br>(1.63)	-0.07<br>(0.09)<br>0.430
Prosocial preferences (hypothetical scenarios)	1271	0.93<br>(0.27)	-0.02<br>(0.02)<br>0.306

		Personal agency	Relational agency
**Outcome**	df	**Mean**<br>(SD)	**Coefficient**<br>(SE)<br>Robust *p*-value
Amina (role model) evaluation	1271	9.56<br>(0.79)	-0.05<br>(0.05)<br>0.335

Note: This data was collected exclusively among the treatment groups immediately post-intervention delivery. No data was collected among the control group. Columns 3 and 4 show regression output comparing the *personal agency* and *relational agency* conditions to each other in Study 3. Composite indices are computed by taking the average of the standardized subcomponents. Regressions control for randomization strata and standard errors are robust. $^{\dagger}p<.10$, *p<.05, **p<.01, ***p<.001.

Table S9. Impacts of varying within-village saturation of treatment (personal or relational agency interventions) on psychosocial outcomes at endline in Study 3

		**Reference group: 25% Saturation of Program Beneficiaries Treated**	**50% Saturation of Program Beneficiaries Treated vs 25%**	**75% Saturation of Program Beneficiaries Treated vs 25%**
**Outcome**	**df**	**Mean (SD)**	**Coefficient (SE) Cluster robust *p*-value**	**Coefficient (SE) Cluster robust *p*-value**
**Psychosocial Composite Index**	**2487**	**-0.02 (1.02)**	**0.08 (0.09) 0.382**	**0.15 (0.09) 0.087†**
**Personal Composite Index**	**2487**	**-0.03 (1.07)**	**0.13 (0.09) 0.145**	**0.14 (0.08) 0.075†**
Well-being	2487	-0.03 (1.04)	0.07 (0.08) 0.390	0.14 (0.08) 0.086†
Self-Efficacy	2473	-0.02 (1.00)	0.05 (0.09) 0.570	0.04 (0.08) 0.636
Future Expectations	2473	-0.03 (1.04)	0.15 (0.08) 0.079†	0.13 (0.08) 0.079†
**Relational Composite Index**	**2487**	**0.00 (0.99)**	**0.03 (0.09) 0.749**	**0.11 (0.08) 0.176**
Partner Dynamics	2226	0.06 (1.01)	-0.03 (0.06) 0.674	0.01 (0.07) 0.906
Household Dynamics	2487	-0.01 (0.99)	0.04 (0.08) 0.648	0.14 (0.08) 0.087†
Control over Earnings	2473	-0.02 (1.03)	-0.03 (0.08) 0.693	0.05 (0.07) 0.430
Social Standing	2473	-0.02 (1.04)	0.06 (0.09) 0.522	0.10 (0.09) 0.270

Social Support	2487	0.00<br>(0.98)	0.07<br>(0.07)<br>0.323	0.08<br>(0.07)<br>0.202
Social Cohesion	2473	-0.01<br>(0.98)	-0.01<br>(0.08)<br>0.870	-0.02<br>(0.08)<br>0.840

Note: Columns 4 and 5 reflect regression output comparing the outcomes of the villages in which respectively 50% or 75% compared to 25% of the village was treated with either the *personal agency* and *relational agency* intervention. Outcomes are standardized to the control condition, at the individual level. Regressions control for randomization strata, and standard errors are clustered at the village level.
†p<.10, *p<.05, **p<.01, ***p<.001.

Table S10. Impacts of varying within-village saturation of treatment (personal or relational agency interventions) on economic outcomes at endline in Study 3

		**Reference group: 25% Saturation of Program Beneficiaries Treated**	**50% Saturation of Program Beneficiaries Treated vs 25%**	**75% Saturation of Program Beneficiaries Treated vs 25%**
**Outcome**	df	Mean (SD)	**Coefficient** (SE) Cluster robust *p*-value	**Coefficient** (SE) Cluster robust *p*-value
**Economic Composite Index**	**2474**	**-0.03 (0.95)**	**0.10 (0.12) 0.382**	**0.11 (0.12) 0.362**
**Food Security Index**	**2473**	**-0.03 (0.97)**	**0.12 (0.11) 0.274**	**0.09 (0.11) 0.427**
Food security	2473	6.86 (1.52)	0.11 (0.14) 0.446	0.06 (0.13) 0.630
Dietary diversity	2473	8.48 (8.13)	0.13 (0.94) 0.232	0.90 (1.00) 0.370
**Business Omnibus Index**	**2474**	**-0.03 (0.95)**	**0.05 (0.10) 0.640**	**0.09 (0.10) 0.388**
**Business Engagement Index**	**2474**	**-0.03 (0.94)**	**0.05 (0.09) 0.583**	**0.10 (0.10) 0.315**
Has a business	2474	0.83 (0.38)	0.01 (0.03) 0.798	0.00 (0.03) 0.866
No. businesses	2474	1.19 (0.96)	0.05 (0.09) 0.578	0.12 (0.10) 0.248
No. businesses past year	2474	0.44 (0.79)	0.06 (0.05) 0.225	0.13 (0.06) 0.039*
Business investments (yearly, USD)	2474	100.53 (178.05)	21.65 (20.15) 0.283	18.51 (19.66) 0.347

Business assets value (yearly, USD)	2474	15.90 (23.15)	-0.88 (1.75) 0.618	-0.59 (1.69) 0.727
No. days worked	2474	16.05 (18.36)	1.24 (1.89) 0.511	2.21 (1.76) 0.209
Growth intentions	2474	1.05 (0.81)	0.01 (0.07) 0.886	0.03 (0.07) 0.653
Healthy business practices index	2457	-0.02 (1.01)	0.01 (0.08) 0.914	0.10 (0.07) 0.169
**Business Performance Index**	**2474**	**-0.01 (0.95)**	**0.02 (0.10) 0.856**	**0.04 (0.10) 0.721**
Business profits (monthly, USD)	2474	34.77 (47.69)	**1.36 (4.80) 0.777**	**2.04 (4.88) 0.676**
Business revenues (monthly, USD)	2474	117.32 (156.89)	1.31 (16.16) 0.935	4.50 (15.67) 0.774

Note: Columns 4 and 5 reflects regression output comparing the outcomes of the villages in which respectively 50% or 75% compared to 25% of the village was treated with either the *personal agency* and *relational agency* intervention. Outcomes are standardized to the control condition, at the individual level. Regressions control for randomization strata, and standard errors are clustered at the village level. †p<.10, *p<.05, **p<.01, ***p<.001.

SI References

1. C. C. Thomas, S. D. Rathod, M. J. De Silva, H. A. Weiss, V. Patel, The 12-item WHO Disability Assessment Schedule II as an outcome measure for treatment of common mental disorders. *Glob. Ment. Health* **3**, e14 (2016).

2. A. Osei-Tutu, *et al.*, Cultural Models of Well-Being Implicit in Four Ghanaian Languages. *Front. Psychol.* **11**, 1798–1798 (2020).

3. A. Bandura, *Self-Efficacy: The Exercise of Control* (Macmillan, 1997).

4. D. Operario, N. E. Adler, D. R. Williams, Subjective social status: reliability and predictive utility for global health. *Psychol. Health* **19**, 237–246 (2004).

5. G. Adams, The Cultural Grounding of Personal Relationship: Enemyship in North American and West African Worlds. *J. Pers. Soc. Psychol.* **88**, 948–968 (2005).

6. A. Aron, E. N. Aron, D. Smollan, Inclusion of Other in the Self Scale and the structure of interpersonal closeness. *J. Pers. Soc. Psychol.* **63**, 596–612 (1992).

7. C. Lund, *et al.*, "The Effects of Mental Health Interventions on Labor Market Outcomes in Low- and Middle-Income Countries" (NBER, 2024).

8. K. L. Milkman, *et al.*, A megastudy of text-based nudges encouraging patients to get vaccinated at an upcoming doctor's appointment. *Proc. Natl. Acad. Sci.* **118** (2021).

9. D. S. Yeager, *et al.*, Teaching a lay theory before college narrows achievement gaps at scale. *Proc. Natl. Acad. Sci.* **113** (2016).

10. E. L. Paluck, R. B. Cialdini, "Field Research Methods" in *Handbook of Research Methods in Social and Personality Psychology*, 2nd Ed., (Cambridge University Press, 2014), pp. 81–98.

11. M. Anderson, J. Magruder, "Split-Sample Strategies for Avoiding False Discoveries" (National Bureau of Economic Research, 2017).

12. J. R. Kling, J. B. Liebman, L. F. Katz, Experimental Analysis of Neighborhood Effects. *Econometrica* **75**, 83–119 (2007).

13. T. J. Ballard, A. W. Kepple, C. Cafiero, "The Food Insecurity Experience Scale: Development of a Global Standard for Monitoring Hunger Worldwide" (FAO, 2013).

14. WFP, "Food Consumption Analysis: Calculation and Use of the Food Consumption Score in Food Security Analysis" (WFP, 2008).

15. R. Schwarzer, M. Jerusalem, "Generalized Self-Efficacy scale" in *Measures in Health Psychology: A User's Portfolio. Causal and Control Beliefs*, J. Weinman, S. Wright, M. Johnston, Eds. (NFER-NELSON, 1995), pp. 35–37.

16. M. Rosenberg, *Conceiving the Self* (Basic Books, 1979).

17. M. L. Ritterman Weintraub, L. C. H. Fernald, N. Adler, S. Bertozzi, S. L. Syme, Perceptions of Social Mobility: Development of a New Psychosocial Indicator Associated with Adolescent Risk Behaviors. *Front. Public Health* **3** (2015).

18. A. Abadie, S. Athey, G. W. Imbens, J. M. Wooldridge, When Should You Adjust Standard Errors for Clustering? *Q. J. Econ.* **138**, 1–35 (2022).

19. T. Bossuroy, *et al.*, Tackling psychosocial and capital constraints to alleviate poverty. *Nature* **605**, 291–297 (2022).
